# A shared, stochastic pathway mediates exosome protein budding along plasma and endosome membranes

**DOI:** 10.1016/j.jbc.2022.102394

**Published:** 2022-08-18

**Authors:** Francis K. Fordjour, Chenxu Guo, Yiwei Ai, George G. Daaboul, Stephen J. Gould

**Affiliations:** 1 Department of Biological Chemistry, Johns Hopkins University, Baltimore, Maryland, USA; 2 Nanoview Biosciences, Boston, Massachusetts, USA

**Keywords:** protein budding, plasma membrane, extracellular vesicle, CD9, CD63, CD81, interferometric reflectance, Rab27a, cDNA, complementary DNA, CTCS, clarified tissue culture supernatant, EV, extracellular vesicle, gDNA, genomic DNA, gRNA, guide RNA, IB, immunoblot, IFM, immunofluorescence microscopy, NTA, nanoparticle tracking analysis, PM, plasma membrane, RPS, resistive pulse sensing, SCC, single cell clone, SPIR, single particle interferometric reflectance, TBST, Tris-buffered saline with Tween-20

## Abstract

Exosomes are small extracellular vesicles of ∼30 to 150 nm that are secreted by all cells, abundant in all biofluids, and play important roles in health and disease. However, details about the mechanism of exosome biogenesis are unclear. Here, we carried out a cargo-based analysis of exosome cargo protein biogenesis in which we identified the most highly enriched exosomal cargo proteins and then followed their biogenesis, trafficking, and exosomal secretion to test different hypotheses for how cells make exosomes. We show that exosome cargo proteins bud from cells (i) in exosome-sized vesicles regardless of whether they are localized to plasma or endosome membranes, (ii) ∼5-fold more efficiently when localized to the plasma membrane, (iii) ∼5-fold less efficiently when targeted to the endosome membrane, (iv) by a stochastic process that leads to ∼100-fold differences in their abundance from one exosome to another, and (v) independently of small GTPase Rab27a, the ESCRT complex–associated protein Alix, or the cargo protein CD63. Taken together, our results demonstrate that cells use a shared, stochastic mechanism to bud exosome cargoes along the spectrum of plasma and endosome membranes and far more efficiently from the plasma membrane than the endosome. Our observations also indicate that the pronounced variation in content between different exosome-sized vesicles is an inevitable consequence of a stochastic mechanism of small vesicle biogenesis, that the origin membrane of exosome-sized extracellular vesicles simply cannot be determined, and that most of what we currently know about exosomes has likely come from studies of plasma membrane-derived vesicles.

Exosomes are secreted, single membrane–bound vesicles that have the same topology as the cell and a diameter of ∼30 to 150 nm (1, 2, 3). Exosomes are enriched in specific subsets of proteins, lipids, and nucleic acids, whereas the larger microvesicles (>0.2 nm diameter) show little evidence for macromolecular enrichment (1, 4, 5). Together, exosomes and microvesicles represent the two major classes of extracellular vesicles (EVs) that are released by normal, uninfected cells, as most other types of EVs are released by damaged, dying, dead, and/or infected cells (*e.g.*, microsomes generated by physical tears in the plasma membrane (PM), apoptotic blebs, oncosomes, enveloped viruses, virus-like particles, etc.) (1). It should be noted that some investigators use the term ‘exosome’ only for vesicles shown definitively to have arisen from endosomes (6, 7). However, we do not see the logic of such a definition (1), in part because there is no way to ever know where any individual exosome arose once it has left the cell, and in part because the data of this paper show there is no substantive difference between small EVs that bud from plasma or endosome membranes.

Exosomes are made by all cells and are present in all biofluids, including blood, urine, cerebrospinal fluid, saliva, amniotic fluid, milk, sweat, bile, vitreous, synovial fluid, bronchial lavage fluid, pleural effusions, and ascites (8, 9, 10, 11, 12, 13, 14, 15, 16, 17). Exosomes contribute to numerous basic cell biological processes, especially protein quality control (18, 19, 20, 21), formation and modulation of extracellular matrix (22, 23, 24, 25), and intercellular transfer of signals and molecules (26, 27, 28, 29, 30, 31, 32, 33, 34, 35, 36, 37, 38, 39). As a signaling particle, exosomes are conceptually distinct from single soluble ligands in that they have the capacity to simultaneously engage and cluster multiple copies of multiple receptors and thereby deliver signals that are unique in mode and tone (1). Exosomes also represent a distinct mechanism by which proteins, lipids, and RNAs can be transferred from one cell to another and perhaps the only way that this can be done over distances in space and time (1).

Given their ubiquity in extracellular fluids (8) and extracellular matrices (23, 25), organisms have evolved to use exosomes in many physiological processes, including fertilization (18, 26, 40), development (40, 41, 42, 43), blood homeostasis (19, 20, 44), bone formation (22, 23, 45), immunity (31, 46, 47, 48), neuronal function (49), and many others (1, 50). Exosomes are also co-opted in many diseases, most obviously in viral infections (2, 51, 52, 53, 54, 55) but also in cancers (30, 56, 57, 58), neuronal disorders (59, 60), and inflammatory diseases (61, 62, 63). Given their broad biomedical relevance, elucidating the molecular mechanisms of exosome biogenesis is likely to have significant impacts on human health and disease.

In light of the broad biomedical importance of exosomes, it is unfortunate that we still do not know how cells (i) recognize exosome cargoes, (ii) direct them to and into nascent, budding vesicles, and (iii) release the resulting exosome-sized vesicles into the extracellular milieu. Our dearth of understanding in these areas reflects a complex biology that simply does not conform to the core principles that characterize other organelle biogenesis pathways.

For example, exosomal cargo proteins do not share a common peptide motif that targets them into exosomes (1, 64, 65, 66, 67). While a combination of PM binding and high order oligomerization are sufficient to target virtually any protein into exosomes (1, 64, 65, 66, 68, 69, 70), the underlying molecular mechanism for this process is still unknown. As for the few exosome ‘targeting signals’ that have been reported, these so far appear to be cargo-specific rather than general. Moreover, they share the property of tethering proteins directly or indirectly to an exosome membrane protein (71, 72, 73), leaving unresolved the question of how the anchoring membrane protein is targeted to exosomes.

Our understanding of *trans*-acting exosome biogenesis factors is similarly limited. For example, numerous lines of evidence implicate the ESCRT machinery in exosome biogenesis (74, 75, 76, 77, 78). Intriguingly, ablation of ESCRT function by expressing dominant-negative forms of VPS4 had no effect on exosome production in multiple cell types in at least two studies (64, 79), yet was found to inhibit exosome cargo and vesicle release over short exosome collection periods of a few hours (78). Thus, there is currently no consensus on how ESCRTs impact the budding of exosome cargo proteins or the release of exosome-sized vesicles from the cell. Other factors thought to play a role in exosome biogenesis include Rab27a, Alix, and CD63, but here again the molecular mechanisms underlying their role(s) in exosome biogenesis remain obscure (77, 80, 81, 82, 83, 84, 85, 86).

The dearth of mechanistic understanding of exosome biogenesis raises the question of whether the field is laboring under a flawed or incomplete operating paradigm (87). Here, we explored this possibility by applying a cargo-based approach similar to those used for studies of other organelle biogenesis systems (88, 89, 90, 91, 92, 93, 94, 95, 96, 97, 98, 99). Specifically, we identified the most highly enriched exosome cargo proteins and then followed their intracellular trafficking and vesicular secretion. The results of our study shed new light on the budding of exosome cargo proteins and exosomes from the cell, and especially the major role of the PM in the secretion of proteins in exosome-sized vesicles.

## Results

### CD9, CD63, and CD81 are the most highly enriched exosome cargo proteins

**Figure 1:**
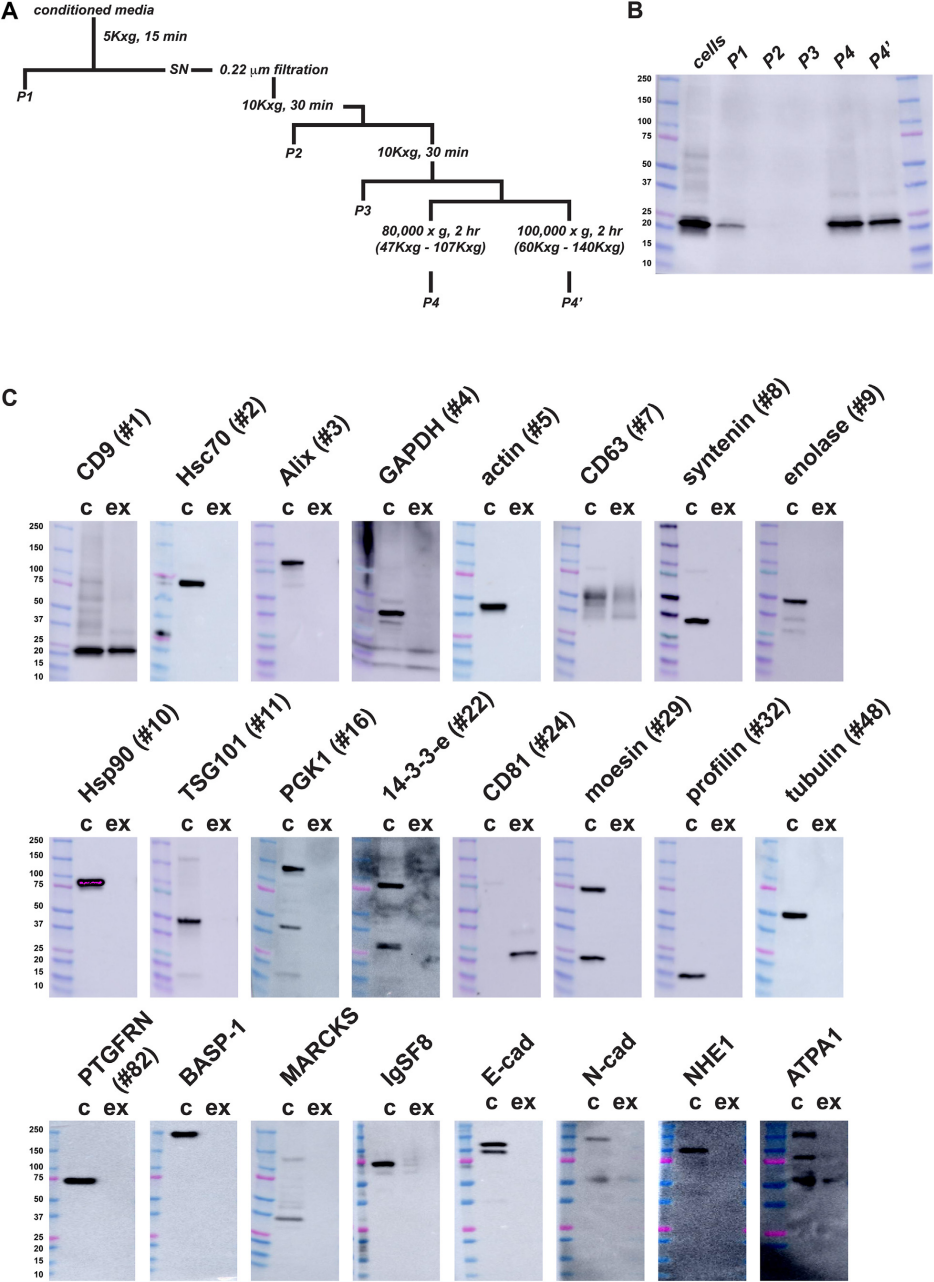
**CD9, CD63, and CD81 are the most highly enriched proteins of HEK293 exosomes.***A*, flow diagram of exosome purification procedure. *B*, anti-CD9 immunoblot of (*left to right*) the 300*g* cell pellet (cells), the 5000*g* pellet (P1), the first 10,000*g* pellet (P2), the second 10,000*g* pellet (P3), and exosome pellets obtained by centrifugation at either 80,000*g* (P4) or 100,000*g* (P4′). *C*, immunoblot analysis of HEK293 cell (c) and exosome lysates (ex), probed with antibodies specific for known exosomal proteins. Molecular weights of size markers are listed to the *left*. Numbers in parentheses refer to the rank on the exocarta.org (150) list of ‘exosome marker proteins’ as of January 1, 2022. CD9, CD63, and CD81 were were ranked as the #1, #7, and #24 most commonly cited exosome marker proteins but were the only proteins that showed obvious enrichment in exosomes. Note also that syntenin (#8), which has recently been suggested to be the most abundant protein in exosomes (151), did not show a level of enrichment that matched that of CD9, CD81, or CD62. These experiments were repeated a minimum of two times for each protein, whereas for other proteins they reflect the results from dozens of independent trials (*e.g*., CD63, CD9, CD81, and Hsp90).

We began our cargo-based analysis of exosome biogenesis by identifying the most highly enriched exosome cargo proteins in a model human cell line, HEK293. Of the many cargo proteins that have been reported, CD9, CD63, and CD81 were the first proteins shown to be enriched in exosomes (46, 100) and as a result have been used as exosome marker proteins for more than 30 years (101, 102, 103). To determine whether these three proteins are more highly enriched than other proteins that have since been reported in exosomes, we collected cell and exosome fractions from HEK293 cultures (Fig. 1, *A* and *B*) and processed them for immunoblot (IB) using antibodies specific for 24 different exosomal proteins. Cell and exosome fractions were loaded at a 1:6 ratio (by proportion of the total cell and exosome fractions) and probed using antibodies specific for CD9 (46), HSC70 (46, 104), Alix (105), GAPDH (106), actin (107), CD63 (100), syntenin (105), enolase (108), HSP90 (104, 109), TSG101 (110), PGK1 (111), 14-3-3 epsilon (112), CD81 (100), moesin (107), profilin (105), tubulin (107), PTGFRN (113), BASP-1 (114), MARCKS (115), IgSF8 (116), E-cadherin (117), N-cadherin (118), NHE1 (119), and ATPA1 (120). All 24 of these proteins were expressed at detectable levels in HEK293 cells, but of these, the most highly enriched proteins in exosomes were CD9, CD63, and CD81 (Fig. 1*C*) These results should not be interpreted as evidence that the other 21 proteins were not present in exosomes, only that they were not as enriched in exosomes as CD9, CD63, and CD81.

The choice of HEK293 cells as our model system was based on several considerations, including its ease of culture, its extensive prior analysis, its noncancer origin, and its recent emergence as the cell line of choice for human exosome engineering (114, 121, 122, 123, 124, 125). Furthermore, additional experiments showed that CD63, CD9, and CD81 were also highly enriched in exosomes produced by nonimmortalized human mammary fibroblasts and dermal fibroblasts, indicating that the contours of exosome biogenesis in HEK293 cells follows that of normal, non-cancerous human cells (Fig. S1).

### Exosome cargo proteins bud more efficiently from plasma than endosome membranes

**Figure 2:**
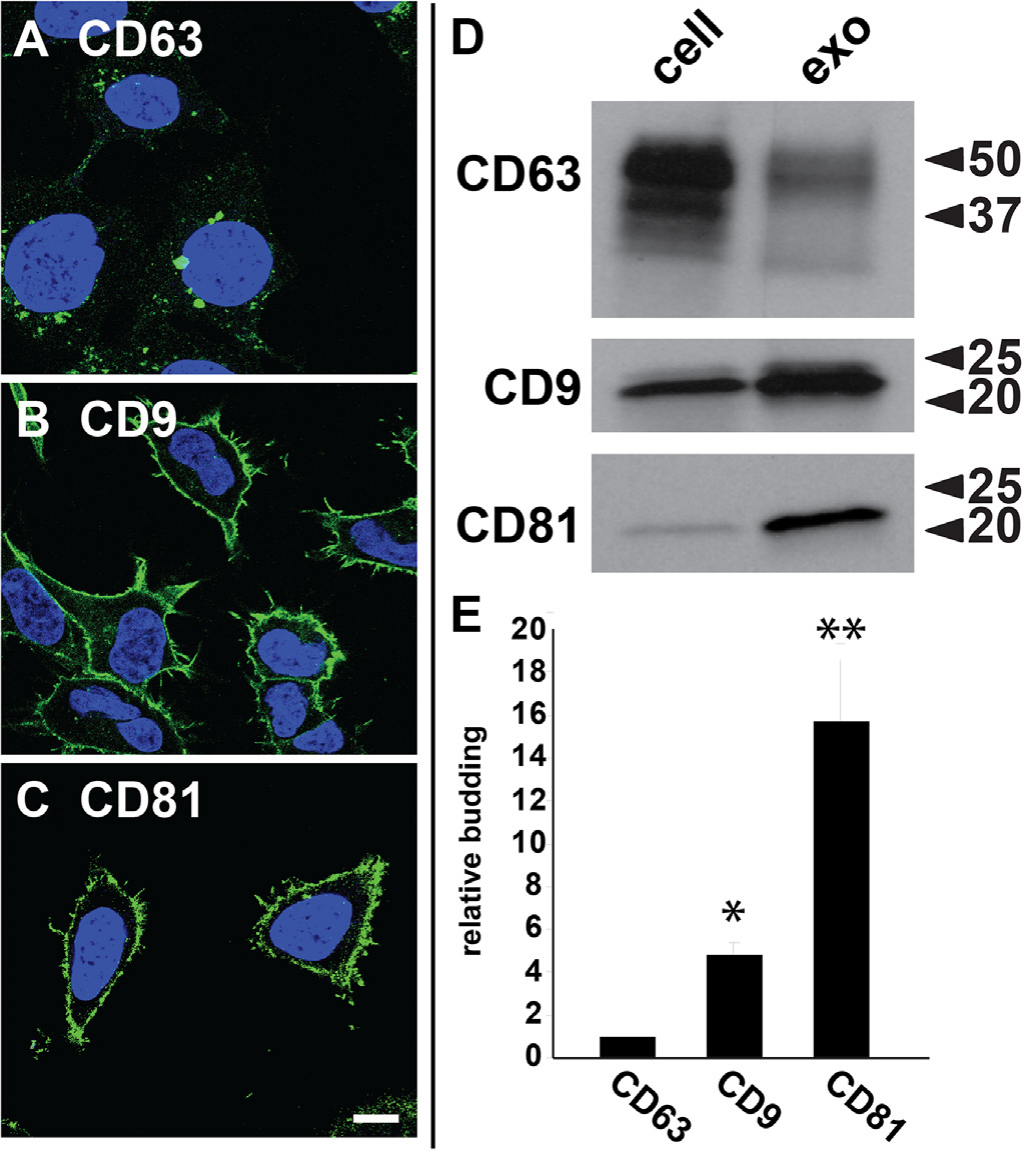
**Plasma membrane–localized exosome cargoes bud more efficiently than an endosome-localized exosome cargo.***A*–*C*, confocal fluorescence micrographs of HEK293 cells that had been fixed, permeabilized, stained with DAPI, and processed for immunofluorescence microscopy using mAbs specific for (*A*) CD63, (*B*) CD9, and (*C*) CD81. Bar, 10 μm. These images are representative representations of more than 100 individual cells examined. *D*, immunoblot of HEK293 cell and exosome fractions probed with antibodies specific for CD63, CD9, and CD81. *E*, bar graph of the relative budding of CD63, CD9, and CD81, with bar height representing the average and error lines denoting the SEM, normalized to that of CD63. The differences in budding relative to CD63 were 4.9-fold for CD9 (±0.53; *p* = 0.0053, n = 4) and 15.7-fold for CD81 (±2.9; *p* = 0.015; n = 4). ∗ and ∗∗ denote *p* values <0.05 and <0.005, respectively, while n = number of trials. DAPI, 4′,6-diamidino-2-phenylindole.

It is widely believed that exosomes are generated by an endosome-dependent pathway of biogenesis. Although confocal immunofluorescence microscopy (IFM) confirmed that CD63 was enriched in endosomes, it also showed that CD9 and CD81 were instead localized to the PM (Fig. 2, *A*–*C*). To determine the relative budding of these proteins, we collected cell and exosome fractions from quadruplicate cultures of HEK293 cells, processed them for IB using antibodies specific for CD63, CD9, and CD81, then calculated their relative budding (relative budding = [amount of protein in exosomes]/[amount of protein in cells + amount of protein in exosomes]) (Fig. 2, *D* and *E*). These experiments revealed that CD63 displayed the lowest relative budding of the three, even though it was the only protein that localized to the endosome. In contrast, the PM-localized CD9 and CD81 proteins displayed relative budding values that were significantly higher than that of CD63: ∼5-fold (4.9 ± 0.53; *p* = 0.0053, n = 4) and ∼15-fold (15.7 ± 2.9; *p* = 0.015; n = 4) higher for CD9 and CD81, respectively. It is important here to state that we use the term ‘budding’ to refer to protein and vesicle release into the extracellular milieu, and it should not be confused with other vesicle budding events, such as those that lead to the lysosome.

### Redirecting the exosomal marker CD63 to the PM increases its vesicular secretion ∼6-fold

**Figure 3:**
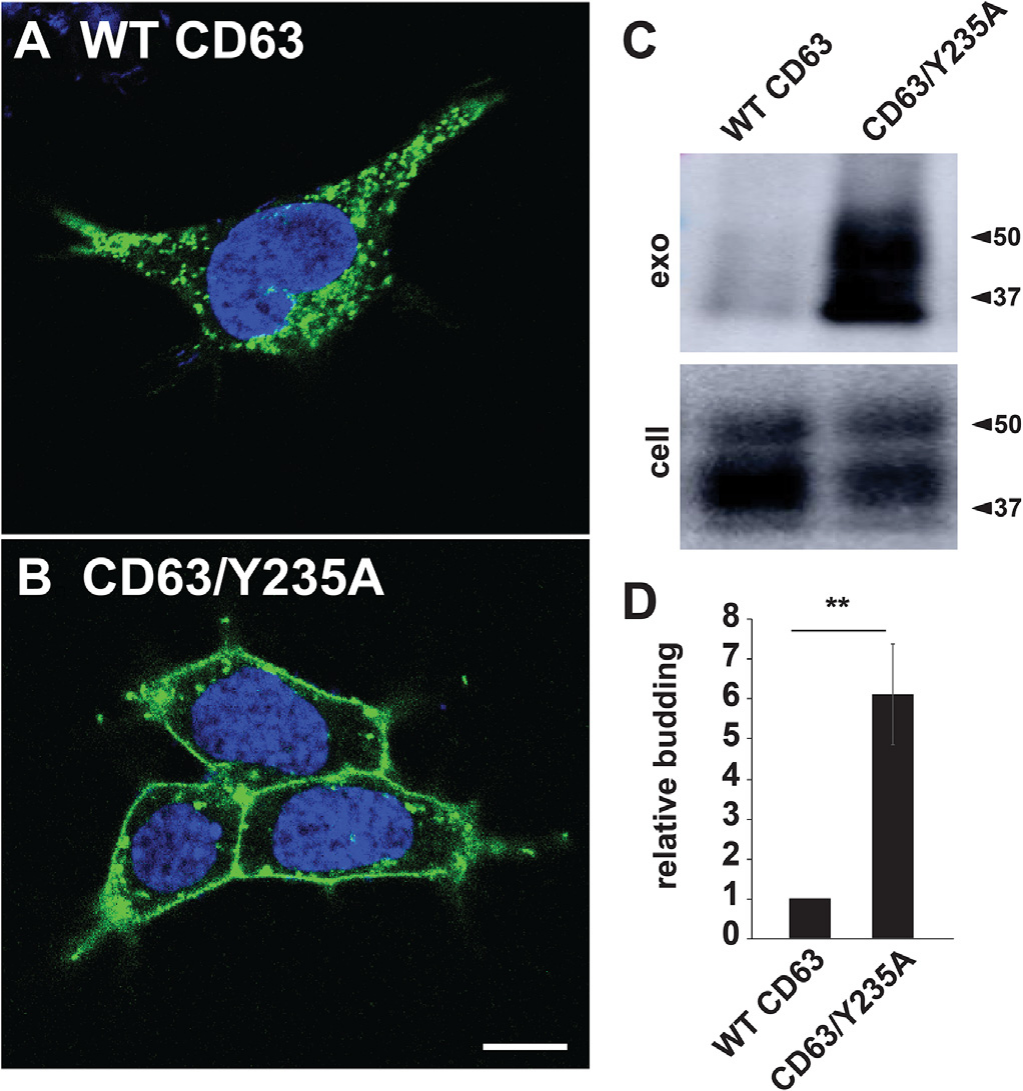
**Redirecting CD63 to the plasma membrane increases rather than decreases its vesicular secretion.***A* and *B*, confocal fluorescence micrographs of CD63^−/−^ cells that had been transfected with plasmids designed to express either (*A*) WT CD63 or (*B*) CD63/Y235A, incubated for 2 days, then fixed, permeabilized, stained with DAPI, and then processed for immunofluorescence microscopy using a mAb specific for CD63. Bar, 10 μm. *C*, anti-CD63 immunoblot of cell and exosome fractions collected from CD63^−/−^ cells expressing either WT CD63 or CD63/Y235A. *D*, bar graph of the relative budding of CD63 and CD63/Y235A, with bar height representing the average and error lines denoting the SEM, normalized to that of CD63. The difference in CD63 budding induced by redirecting CD63 to the plasma membrane was 6.1 ± 1.3-fold (*p* = 0.0038; n = 9). ∗∗ denotes a *p* value <0.005, while n = number of trials.

These observations raise the possibility that the budding of exosome cargo proteins is more efficient from the PM than from the endosome membrane. To test this hypothesis, we asked whether redirecting CD63 from the endosome to the PM would lead to an increase in its secretion from the cell in exosome-sized vesicles. Toward this end, we first created a CD63^−/−^ null cell line using Cas9/guide RNA (gRNA)–mediated gene editing (Fig. S2). This cell line was then transfected with plasmids that expressed either WT CD63 or CD63/Y235A, which lacks the essential tyrosine of its YXXΦ-based endocytosis signal (126). Two days later, cells were examined by (i) confocal IFM to determine their subcellular distributions and (ii) IB of cell and exosome fractions to measure the relative budding of these two forms of CD63. As expected, WT CD63 was targeted to endosomes, whereas CD63/Y235A was localized primarily to the PM (Fig. 3, *A* and *B*). As for their relative budding, IB of cell and exosome fractions showed that CD63/Y235A budded ∼6-fold more efficiently than CD63 (6.1 ± 1.3-fold, *p* = 0.0038; n = 9), establishing that CD63 buds far more efficiently when localized to the PM (Fig. 3, *C* and *D*). To test whether similar results would be observed in other cell types, we repeated these experiments in NIH3T3 cells and obtained similar results (Fig. S3).

**Figure 4:**
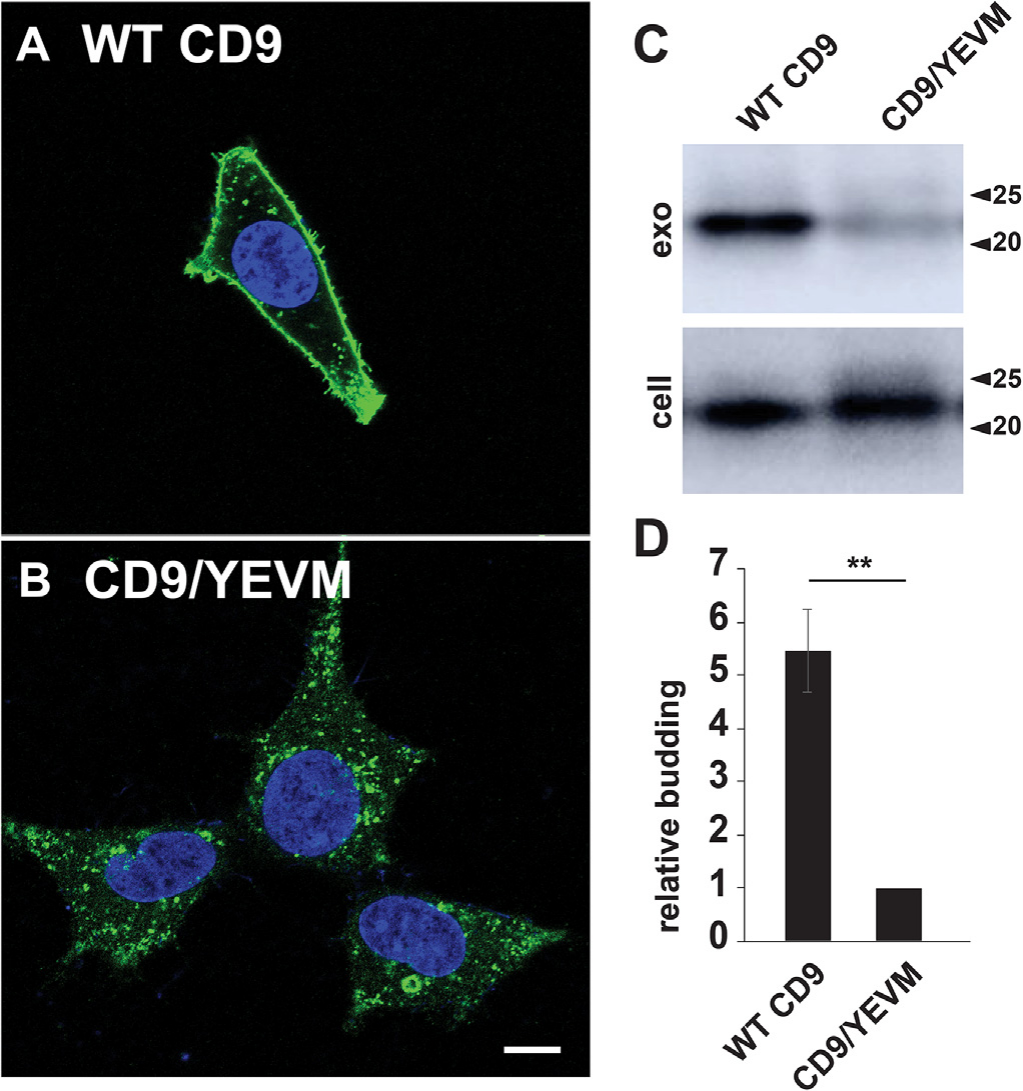
**Redirecting CD9 to endosomes decreases rather than increases its vesicular secretion.***A* and *B*, confocal fluorescence micrographs of CD9^−/−/−^ cells that had been transfected with plasmids designed to express either (*A*) WT CD9 or (*B*) CD9/YEVM, incubated for 2 days, then fixed, permeabilized, stained with DAPI, and then processed for immunofluorescence microscopy using a mAb specific for CD9. Bar, 10 μm. *C*, anti-CD9 immunoblot of cell and exosome fractions collected from CD9^−/−/−^ cells expressing either WT CD9 or CD9/YEVM. *D*, bar graph of the relative budding of CD9 and CD9/YEVM, with bar height representing the average and error lines denoting the SEM, normalized to that of CD9. The magnitude of the decrease in CD9 budding brought on by its targeting to endosomes was 5.5-fold (*p* = 0.00019; n = 9). ∗∗ denotes a *p* value <0.005, while n = number of trials.

**Figure 5:**
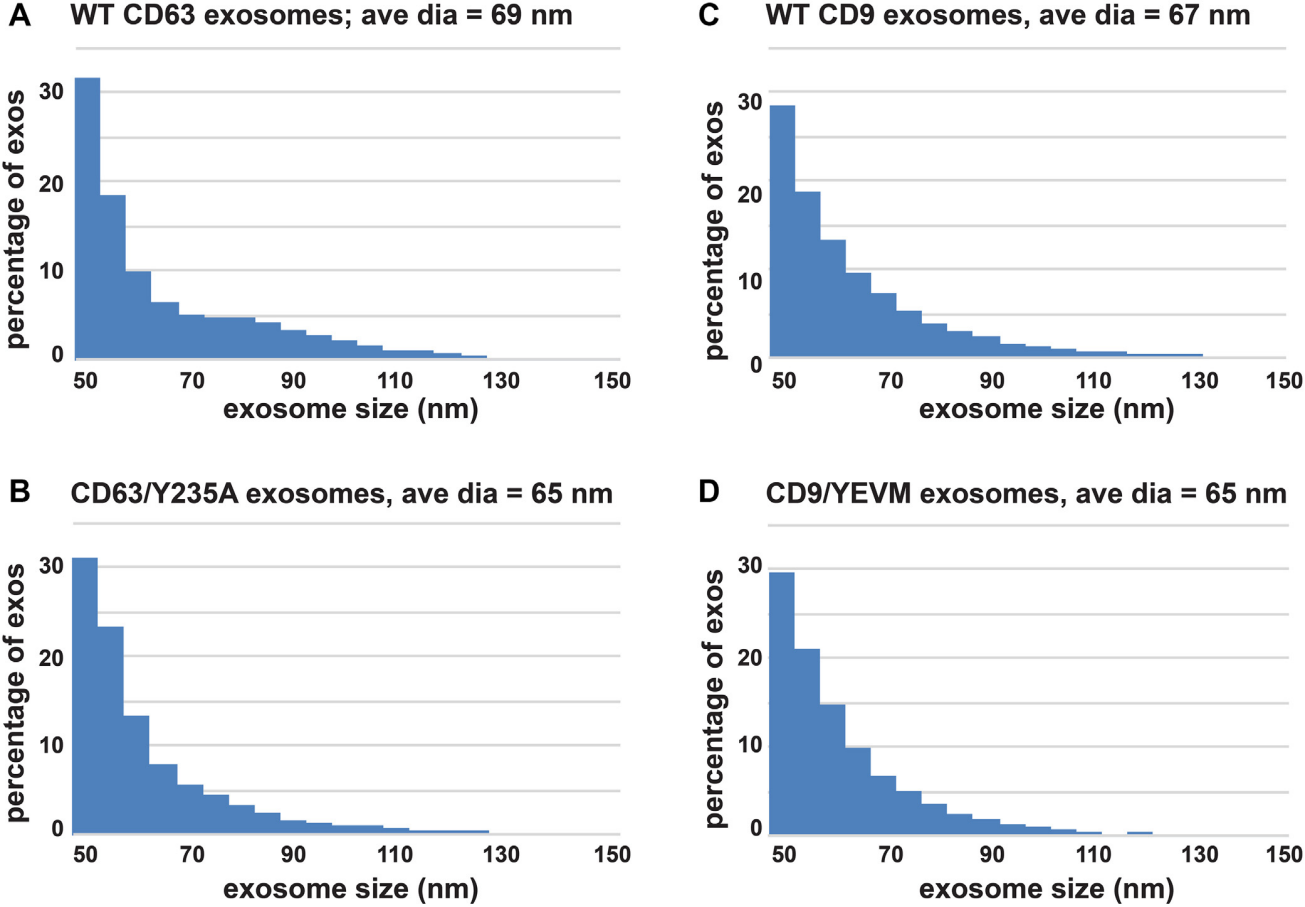
**CD63 and CD9 are found in exosomes of the same size, regardless of whether they are localized to the plasma or endosome membrane.***A* and *B*, histograms showing the diameter of (*A*) CD63-containing exosomes (n = 3686) and (*B*) CD63/Y235A-containing exosomes (n = 5569), as determined by SPIR imaging, with exosome diameter plotted on the *x*-axis against the percentage of exosomes on the *y*-axis that fell within each 5 nm window of sizes. *C* and *D*, histograms showing the diameter of (*C*) CD9-containing exosomes (n = 15,684) and (*D*) CD9/YEVM-containing exosomes (n = 18,323), as determined by SPIR imaging, with exosome diameter plotted on the *x*-axis against the percentage of exosomes on the *y*-axis that fell within each 5 nm window of sizes. This experiment was performed three times. SPIR, single particle interferometric reflectance.

**Figure 6:**
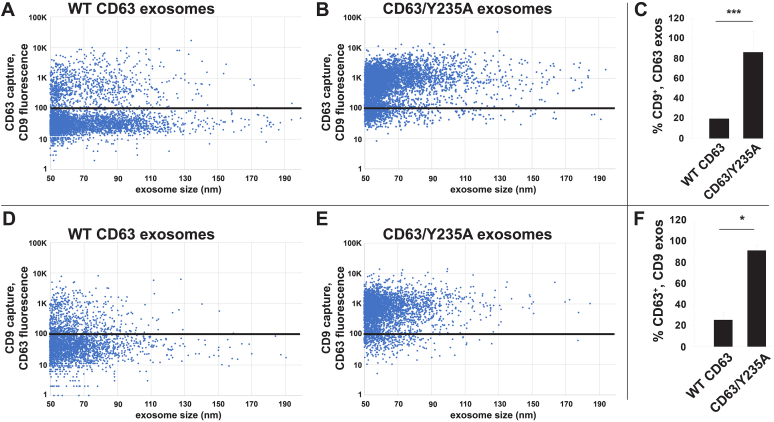
**Redirecting CD63 to the plasma membrane leads to its cobudding into the same exosomes as CD9.***A* and *B*, scatter plots showing exosome size on the *x*-axis and anti-CD9 immunostaining intensity on the *y*-axis, for (*A*) CD63-containing exosomes and (*B*) CD63/Y235A-containing exosomes, as determined by SPIR-IFM of exosomes captured on an anti-CD63-functionalized SPIR imaging chip. The *black horizontal lines* depict the threshold of specific immunostaining, as determined using isotype-specific control antibodies. *C*, bar graph showing that the percentage of CD63-containing exosomes (20% ± 1%; n = 3) and CD63/Y235A-containing exosomes (86% ± 21%; n = 3) that carried CD9 differed by ∼4-fold (*p* = 0.0001; n = 3), with ∗∗∗ denoting the *p* value − 0.0001. *D* and *E*, scatter plots showing exosome size on the *x*-axis and anti-CD63 immunostaining intensity on the *y*-axis, for (*A*) CD63-containing exosomes and (*B*) CD63/Y235A-containing exosomes, as determined by SPIR-IFM of exosomes captured on an anti-CD9-functionalized SPIR imaging chip. The *black horizontal lines* depict the threshold of specific immunostaining, as determined using isotype-specific control antibodies. *F*, bar graph showing that the percentage of CD9-containing exosomes that carried CD63 (25% ± 7%; n = 3) was significantly lower than those that carried CD63/Y235A (91% ± 5%; n = 3), a 3.6-fold difference (*p* = 0.032; n = 3). ∗ denotes a *p* value <0.05, while n = number of trials. IFM, immunofluorescence microscopy; SPIR, single particle interferometric reflectance.

**Figure 7:**
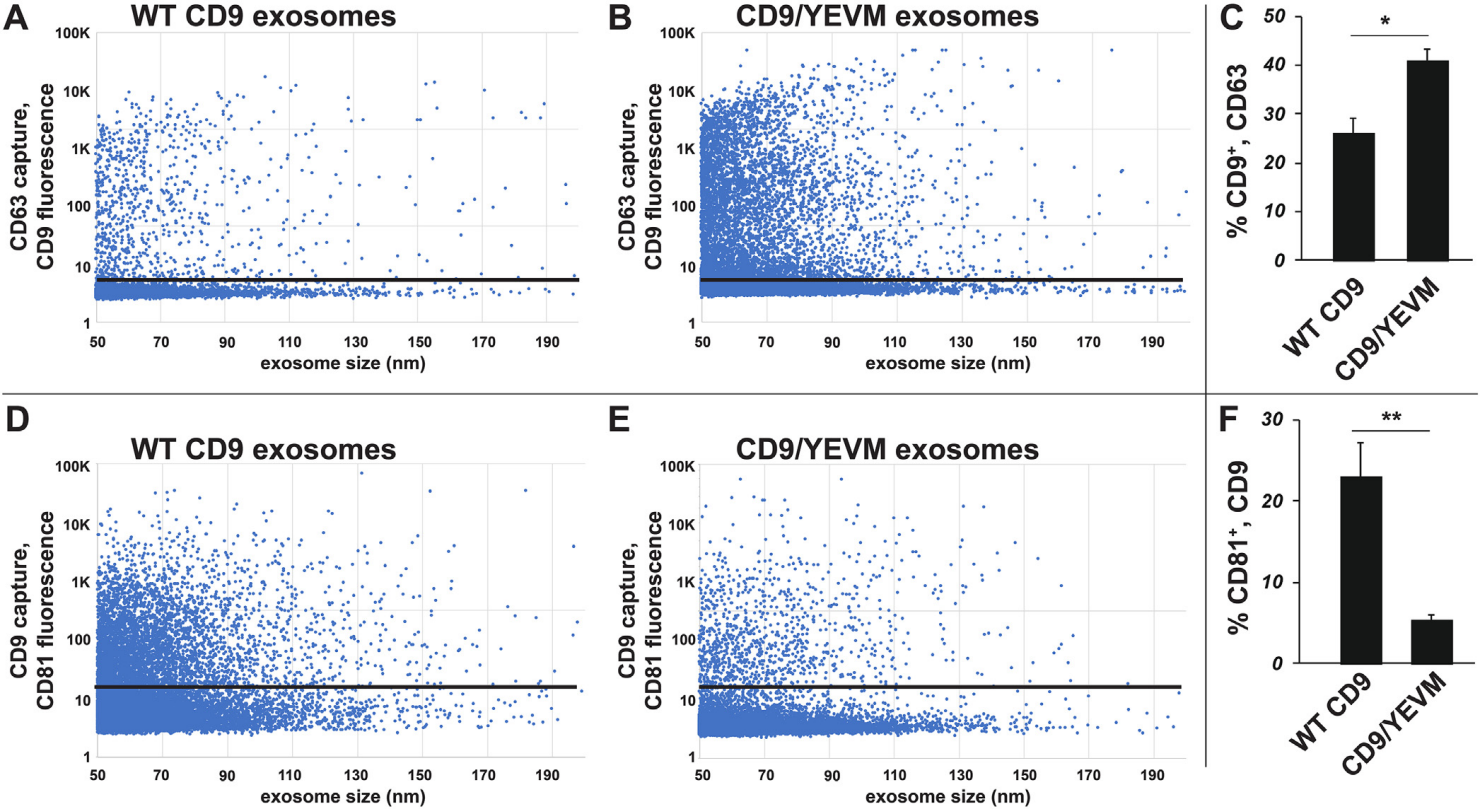
**Redirecting CD9 to endosomes increased its co-budding with CD63 and decreased its cobudding with CD81.***A* and *B*, scatter plots showing exosome size on the *x*-axis and anti-CD9 immunostaining intensity on the. *y*-axis, for (*A*) CD9-containing exosomes and (*B*) CD9/YEVM-containing exosomes, as determined by SPIR-IFM of exosomes captured on an anti-CD63-functionalized SPIR imaging chip. The *black horizontal lines* depict the threshold of specific immunostaining, as determined using isotype-specific control antibodies. *C*, bar graph showing that the percentage of CD63-containing exosomes that contained CD9/YEVM (41% ± 2%; n = 9) was significantly higher (*p* = 0.011) than the percentage of CD63-containing exosomes that contained CD9 (26% ± 3%; n = 9), with ∗∗∗ denoting the *p* value ≤ 0.0001, while n = number of trials. *D* and *E*, scatter plots showing exosome size on the *x*-axis and anti-CD81 immunostaining intensity on the *y*-axis, for (*A*) CD9-containing exosomes and (*B*) CD9/YEVM-containing exosomes, as determined by SPIR-IFM of exosomes captured on an anti-CD9 functionalized SPIR imaging chip. The *black horizontal lines* depict the threshold of specific immunostaining, as determined using isotype-specific control antibodies. *F*, bar graph showing that CD81 was found on a significantly higher percentage of on CD9-containing exosomes (23% ± 4%; n = 9) than it was on CD9/YEVM-containing exosomes (5.4% ± 0.7%; n = 9, *p* = 0.0037). ∗∗ denoting the *p* value ≤ 0.005; n = number of trials. IFM, immunofluorescence microscopy; SPIR, single particle interferometric reflectance.

It should be noted that we publicly disclosed all of the experiments and results presented in Figure 1, Figure 2, Figure 3, Figure 4, Figure 5, Figure 6, Figure 7 of this article in February 2019 on bioRxiv (https://www.biorxiv.org/content/10.1101/545228v1) (127). Furthermore, while similar results to the data presented in our Figures 2 and 3 were reported later by Mathieu *et al*. (https://www.biorxiv.org/content/10.1101/2020.10.27.323766v1) (6, 127).

### Targeting CD9 to endosomes decreases its vesicular secretion

We next performed the corollary experiment, which was to test whether redirecting CD9 from the PM to endosomes led to an increase or decrease in CD9 budding. Using Cas9/gRNA-mediated gene editing, we introduced inactivating mutations into all three alleles of the CD9 gene that are present in HEK293 cells (Fig. S4). The resulting CD9^−/−/−^ HEK293 cell line was then used to assess the subcellular distribution and relative budding of both WT CD9 and CD9/YEVM, a mutant form of CD9 that carries the endosome-targeting signal (YEVM) of CD63 at its C terminus. Confocal fluorescence microscopy confirmed that WT CD9 was localized primarily at the PM, whereas CD9/YEVM was localized to endosomes (Fig. 4, *A* and *B*). Cells and exosomes were collected from these cells, and IB analysis revealed that redirecting CD9 from the cell surface to endosomes caused a 5.5-fold decrease in its vesicular secretion (*p* = 0.00019; n = 9) (Fig. 4, *C* and *D*). Similar results were obtained when these experiments were repeated in NIH3T3 cells, indicating that the reduced budding of endosome–localized cargo proteins is evident in mouse as well as human cell lines (Fig. S5).

### CD63 and CD9 are secreted in exosome-sized vesicles regardless of their intracellular distribution

It has often been asserted that EVs that bud from the PM are larger than those that bud from the endosome membrane (128, 129, 130, 131, 132). We therefore measured the sizes of EVs that contain CD63 or CD63/Y235A. This was done using single particle interferometric reflectance (SPIR) imaging, a label-free optical technique that can measure vesicle sizes in the range of 50 to 200 nm (133, 134). In brief, specialized SPIR imaging chips were functionalized with a mAb specific for CD63, then incubated with exosomes collected from CD63^−/−^ cells expressing either WT CD63 or CD63/Y235A. After the exosomes were immunopurified on the SPIR chip, the diameter of thousands of individual vesicles was determined by SPIR imaging. The results of these experiments revealed that the diameter of CD63/Y235A-containing EVs (65 nm (n = 5569) was similar to that of CD63-containing EVs (69 nm (n = 3686) (Fig. 5, *A* and *B*). Paralogous experiments were performed on EVs prepared from CD9^−/−/−^ cells that expressed either WT CD9 or CD9/YEVM. These were incubated on SPIR imaging chips that had been functionalized with a monoclonal anti-CD9 antibody, washed, and subjected to SPIR imaging. Exosomes containing CD9 had an average diameter of 67 nm (n = 15,684), whereas exosomes containing CD9/YEVM had an average diameter of 65 nm (n = 18,232) (Fig. 5, *C* and *D*). Taken together, these demonstrate that that exosome cargo proteins bud from the PM in exosome-sized vesicles.

### Shared budding I: Redirecting CD63 to the PM increases its cobudding with CD9

The most parsimonious explanation for these observations is that cells use a shared pathway to bud the same exosome cargo proteins from both plasma and endosome membranes in vesicles of the same size and topology. If this hypothesis is correct, then cells should load CD63 and CD9 into the same individual exosomes whenever they are colocalized along the spectrum of plasma and endosome membranes. To test this hypothesis, we coupled SPIR imaging to IFM and measured the amounts of exosome cargo proteins on the surface of exosomes released by cells expressing either WT CD63 or CD63/Y235A. Specifically, we transfected CD63^−/−^ cells with vectors designed to express either WT CD63 or CD63/Y235A, followed by collection of exosomes from the culture medium by filtration, size-exclusion chromatography, and immunopurification on SPIR imaging chips functionalized with an anti-CD63 capture antibody. These immunopurified vesicles were then incubated with a fluorescently tagged, CD9-specific mAb, and the level of anti-CD9 fluorescence was measured for each bound exosome (Fig. 6, *A* and *C*). These experiments revealed that CD9 was present on ∼20% of CD63-containing exosomes (20% ± 1%; n = 3). In contrast, CD9 was detected on >80% of CD63/Y235A-containing exosomes (86% ± 21%; n = 3) (Fig. 6, *B* and *C*). These data show that redirecting CD63 to the PM led to a ∼4-fold increase in its cobudding with CD9 (*p* = 0.00011; n = 3).

Similar results were observed when the same samples were interrogated by capture on SPIR imaging chips functionalized with anti-CD9 antibodies, followed by incubation with a fluorescently tagged anti-CD63 antibody (Fig. 6, *D*–*F*). Specifically, we observed that CD63 was found on ∼25% of CD9-containing exosomes (25% ± 7%; n = 3), whereas CD63/Y235A was found on >90% of CD9-containing exosomes (91% ± 5%), a >3-fold difference (3.6×; *p* = 0.032; n = 3) that suggests that cells use a single, shared mechanism for loading both CD63 and CD9 into identical exosome-sized vesicles.

### Shared budding II: Redirecting CD9 to the endosome increases its cobudding with CD63

We next tested whether redirecting CD9 to endosomes increased its cobudding with WT CD63. Exosomes were collected from CD9^−/−/−^ cells expressing either PM-localized WT CD9 or endosome-localized CD9/YEVM, immunopurified on SPIR imaging chips functionalized with anti-CD63 mAb, and then interrogated with fluorescently tagged anti-CD9 antibodies. These experiments demonstrated that CD9 was present on ∼26% of CD63-positive exosomes (± 3%; n = 9), whereas CD9/YEVM was detected on 41% of CD63-positive exosomes (± 2%; n = 9), a 70% increase in the cobudding of these proteins (*p* = 0.011, n = 9) (Fig. 7, *A*–*C*). These results lend further support to the hypothesis that mammalian cells use the same basic mechanism for loading both CD9 and CD63 into exosome-sized EVs, regardless of whether the nascent vesicles are budding from plasma or endosome membranes.

### Shared budding III: CD9 displays no more cobudding with CD81 than with CD63

A proteomic analysis of antigenically defined exosomes concluded that CD63 defined an endosome-dependent pathway of exosome cargo protein budding, while CD9 defined a distinct, PM-dependent pathway of vesicle biogenesis (6). To query this hypothesis, we tested whether the degree of cobudding between the PM-localized cargoes CD9 and CD81 was greater than the ∼20% to 25% cobudding that we observed above for CD9 and CD63. Specifically, we collected exosomes from CD9^−/−/−^ cells expressing either PM-localized WT CD9 or endosome-localized CD9/YEVM, immunopurified the vesicles on anti-CD9 mAb immobilized on SPIR imaging chips, and then interrogated the vesicles with fluorescently tagged anti-CD81 antibodies. These experiments revealed that CD81 cobudded with CD9 on only 23% of secreted vesicles (±4%; n = 9) (Fig. 7, *D* and *F*). This is roughly the same percentage that we observed previously for CD9 and CD63, indicating that differences in antigenically defined subpopulations can be caused by variables other than membrane of origin or presumed mechanism of biogenesis. As for whether redirecting CD9 to the endosome further reduced the cobudding of CD9 and CD81, we found that it did, as only ∼5% of CD9/YEVM exosomes contained CD81 (5.4% ± 0.7%; n = 9; *p* = 0.0037) (Fig. 7, *E* and *F*).

### Shared budding IV: CD63, CD9, and CD81 levels vary by ≥100-fold from one exosome to another

As noted before, the compositional differences between antigenically defined subpopulations of vesicles have led to the notion that cells generate exosome-sized EVs by self-assembly mechanisms that generate distinct vesicle ‘types’ that reflect different membranes of origin and mechanisms of biogenesis (6). The defining feature of such self-assembly mechanisms is that they will generate nanostructures of relatively consistent composition (135, 136). This is not, however, reflected in our immunophenotyping analysis of individual exosomes, which revealed that CD9, CD63, and CD81 each varied by 100-fold or more from one exosome-sized vesicle to the next (Figs. 6 and 7). This extent of compositional heterogeneity argues strongly that exosome biogenesis is mediated by a stochastic process and shows directly that there is no such thing as a ‘type’ exosome. As for the fact that antigenically defined exosome subsets can have different compositions, these differences are likely idiosyncratic in nature rather than manifestations of distinct mechanisms of cargo protein selection and vesicle budding.

### Exosome protein budding and exosome yield are unaffected by KO of Rab27a or Alix

It is widely presumed that exosome biogenesis requires the small GTPase Rab27a (82), the ESCRT-associated protein Alix (77, 85), and CD63 itself (86). To test whether these genes are required for exosome biogenesis in general, we used CRISPR/Cas9 gene editing to generate KO cell lines for each of these genes and then assayed the resulting mutant cell lines for their vesicular secretion of exosome cargo proteins and their release of exosome-sized vesicles.

**Figure 8:**
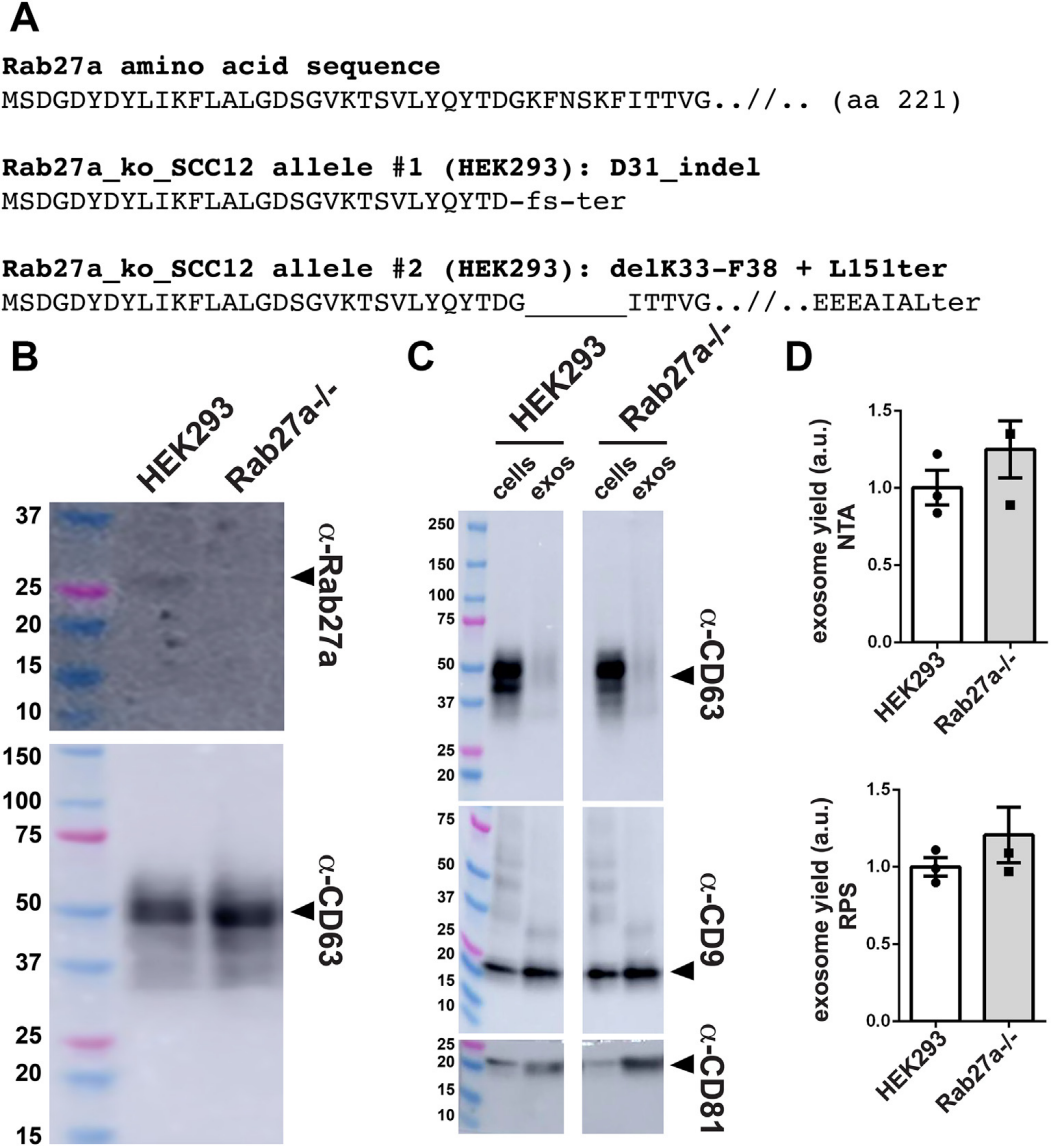
**Exosome biogenesis by HEK293 cells is unaffected by inactivating mutations in the Rab27a gene.***A*, amino acid sequence of (*upper line*) WT Rab27a and (*lower lines*) predicted protein products of Rab27a alleles #1 and #2 in the HEK293 Rab27a^−/−^ cell line Rab27a_ko_SCC12. Allele #1 is the result of an ∼10,000 bp deletion of the DNA between the Cas9 target sites in exon 2 and exon 4, as well as an insertion of undefined length in its place. This large insertion/deletion (indel) mutation removed the splice donor site at the 5′ end of intron 2. Sequence analysis of Rab27a cDNAs from this cell line revealed that this allele expresses an mRNA derived from a cryptic splice donor site within exon 2 spiced to various a splice acceptor sites within intron 3, deleting all of exon 3 in the process. The resulting mRNAs are incapable of expressing more than the first 31 amino acids of the 221 amino acid-long Rab27a protein. Allele #2 carries two mutations, a 12 bp deletion in exon 2 and a frameshift mutation in exon 4. The exon 4 mutation deletes the C-terminal 70 amino acids of the protein, including its GTP-binding pocket (152, 153) and C-terminal cysteine residues required for its prenylation, membrane localization, and function (154), while the in-frame exon 2 deletion eliminates the unique peptide insertion that defines the Rab27 protein family. *B*, immunoblot analysis of whole cell lysates of Rab27^−/−^ cells and WT HEK293 cells, interrogated with antibodies specific for (*upper panel*) Rab27a and (*lower panel*) CD63. Arrowheads denote the detected proteins. Molecular weight markers in kDa are shown to the right. *C*, immunoblot of cell and exosome fractions collected from cultures of WT and Rab27a^−/−^ cell lines, probed with antibodies specific for CD63, CD9, and CD81. Molecular weight markers are shown in kDa. *D*, bar graphs of exosome yield of triplicate cultures of WT and Rab27a^−/−^ cells, as determined by (*upper graph*) nanoparticle tracking analysis and (*lower graph*) resistive pulse sensing. Bar height denotes the average, error bars represent the SEM, and individual data points are shown. These experiments were performed three times. cDNA, complementary DNA.

To mutate the Rab27a gene, we transfected HEK293 cells with pJM1064, a Cas9-expressing vector designed to also express a pair of gRNAs that target sites in exon 2 and exon 4 of the Rab27a gene, as well as EGFP, herpes simplex virus thymidine kinase (tk), and PuroR, each expressed from the same polycistronic mRNA as Cas9, with the entirety of this ORF flanked by loxP sites for subsequent Cre-mediated deletion of the Cas9-2a-EGFP-2a-tk-PuroR ORF. Puromycin-resistant cells were selected, single cell clones (SCCs) were isolated, and the SCCs were interrogated by sequencing of genomic DNA (gDNA) in the vicinity of the Rab27a target sites. This effort yielded a Rab27a^−/−^ HEK293 cell line with null mutations on both of its Rab27a alleles. The first of these has a large insertion/deletion (indel) resulting from deletion of most of the DNA between exons 2 and 4. The second allele carries a frameshift mutation in exon 4 that deletes the C-terminal 70 codons and much of the GTP-binding pocket of Rab27a, as well as an in-frame deletion of 6 codons in exon 2 that remove the conserved insert that defines the Rab27 protein family (Fig. 8*A*). Consistent with the DNA sequences of these Rab27a alleles, sequence analysis of Rab27a complementary DNAs (cDNAs) from this cell line established that neither allele expressed an mRNA capable of encoding a functional form of Rab27a protein. Specifically, mRNAs expressed from allele #1 lacked most of exon 2, all of exon 3, and could not express anything more than a short N-terminal peptide of Rab27a, whereas mRNAs expressed from allele #2 lacked the ability to encode the C-terminal 70 amino acids of the protein or the Rab27-specific insert in its N terminus. Consistent with these results, IB analysis of whole cell lysates showed the presence of Rab27a protein in HEK293 cell lysates but not in a lysate of Rab27a^−/−^ cells (Fig. 8*B*). To remove the Cas9, GFP, HSV tk, and PuroR ORF from this cell line, it was transfected with a Cre recombinase-expressing plasmid, followed by selection of HSV tk-deficient cells in media containing ganciclovir, yielding the Rab27a^−/−^ cell line used in further studies.

To test whether Rab27a was required for exosome biogenesis, we next collected cell and exosome fractions from triplicate cultures of WT HEK293 cells and the Rab27a^−/−^ cell line. IB analysis of cell and exosome fractions revealed that loss of Rab27a had no effect on the vesicular secretion of CD63, CD9, or CD81 (Fig. 8*C*). Consistent with these results, we found that Rab27a^−/−^ cells also showed no decrease in exosome yield, regardless of whether it was measured by nanoparticle tracking analysis (NTA) or resistive pulse sensing (RPS) (Fig. 8*D*).

**Figure 9:**
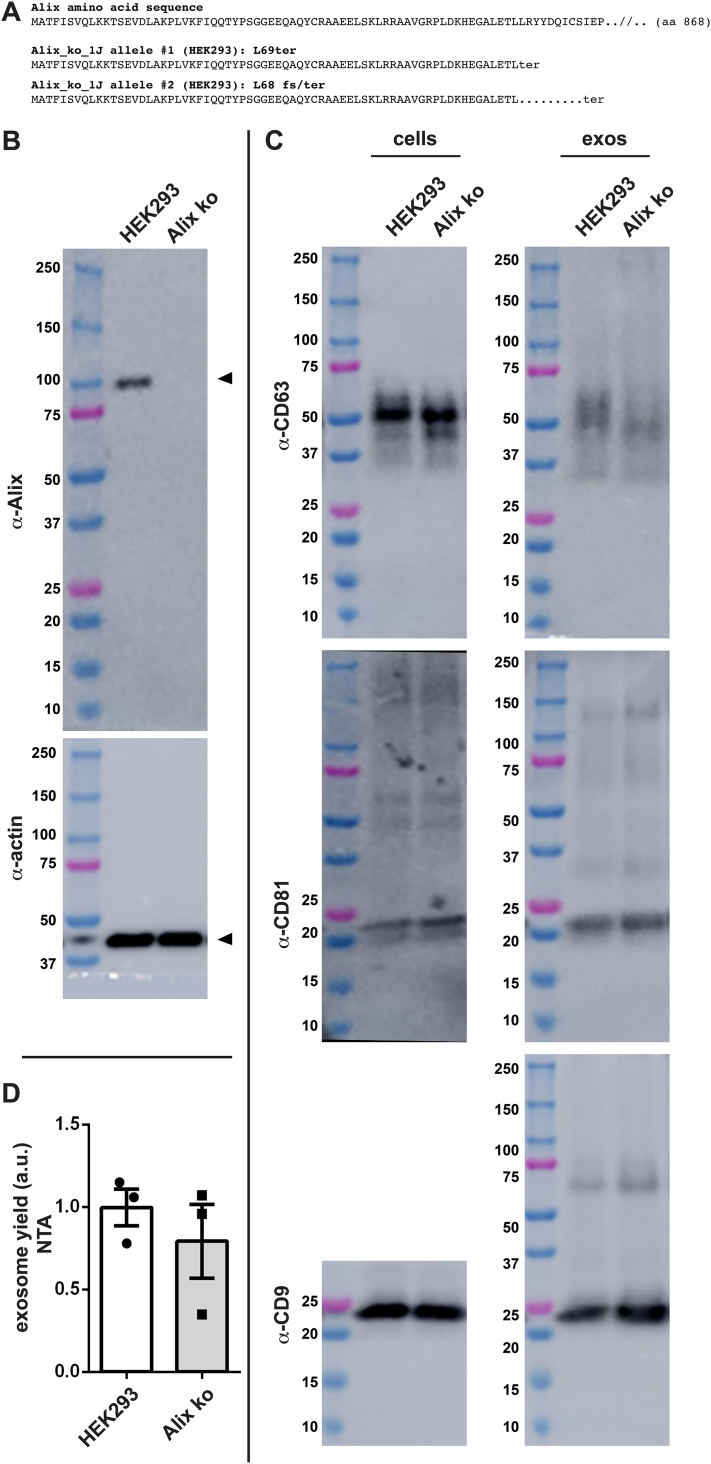
**Exosome biogenesis by HEK293 cells is unaffected by inactivating mutations in the Alix gene.***A*, amino acid sequence of (*upper line*) the WT Alix gene and (*lower lines*) the predicted protein products of Alix alleles #1 and #2 in the HEK293 Alix^−/−^ cell line 1J. Allele #1 carries a stop codon at position 69 of the 868 amino acid-long ORF due to an 85 nucleotide-long insertion into exon 1. Allele #2 carries a ∼38,000 bp deletion between exon 1 and exon 8 that shifts the reading frame after codon 68, leading to a stop codon nine codons later. *B*, immunoblot analysis of whole cell lysates of WT HEK293 cells and the Alix^−/−^ cell line 1J, interrogated with antibodies specific for (*upper panel*) Alix and (*lower panel*) actin. Arrowheads denote the detected proteins. Molecular weight markers are shown in kDa. *C*, immunoblot of cell and exosome fractions collected from cultures of WT HEK293 cells and the Alix^−/−^ cell line 1J, probed with antibodies specific for CD63, CD81, and CD9. *D*, bar graphs of exosome yield of triplicate cultures of WT and Alix^−/−^ cells, as determined by nanoparticle tracking analysis. Bar height denotes the average, error bars represent the SEM, and individual data points are shown. These experiments were performed three times.

The observation that Rab27a^−/−^ cells displayed normal levels of exosome cargo protein budding and exosome release raised the possibility that HEK293 cells use a different ‘endosomal regulator’ in their pathway of exosome biogenesis. Alix is the most obvious candidate, as it represents a potential link between exosome biogenesis and the ESCRT machinery (77, 85). Alix was deleted by the same general approach as described before, this time using the plasmid FF3, which expresses gRNAs that target sites in coding exons 1 and 8 of the Alix gene. This yielded the Alix_ko_1J cell line, which proved to carry inactivating mutations on both of its Alix alleles (Fig. 9*A*). Specifically, allele #1 carried an insertion mutation in exon 1 that replaced the Leu69 codon with a stop codon, whereas allele #2 carried an ∼38,000 bp deletion of all DNA between exon 1 and exon 8, while also introducing a frameshift mutation at codon 69 and a stop codon shortly thereafter. Not surprisingly, this Alix^−/−^ HEK293 cell line lacks detectable expression of Alix protein (Fig. 9*B*).

With this Alix-deficient cell line in hand, we next measured the effect of Alix disruption on the relative budding of CD63, CD9, and CD81. WT and Alix^−/−^ cells were grown in parallel for 3 days, followed by collection of cell and exosome fractions. IB of these fractions revealed that loss of Alix had no substantive effect on the secretion of any of these proteins in exosome-sized vesicles (Fig. 9*C*). Loss of Alix also had no discernable effect on exosome yield, as determined by NTA (Fig. 9*D*).

### KO of CD63 has no effect on exosome biogenesis

**Figure 10:**
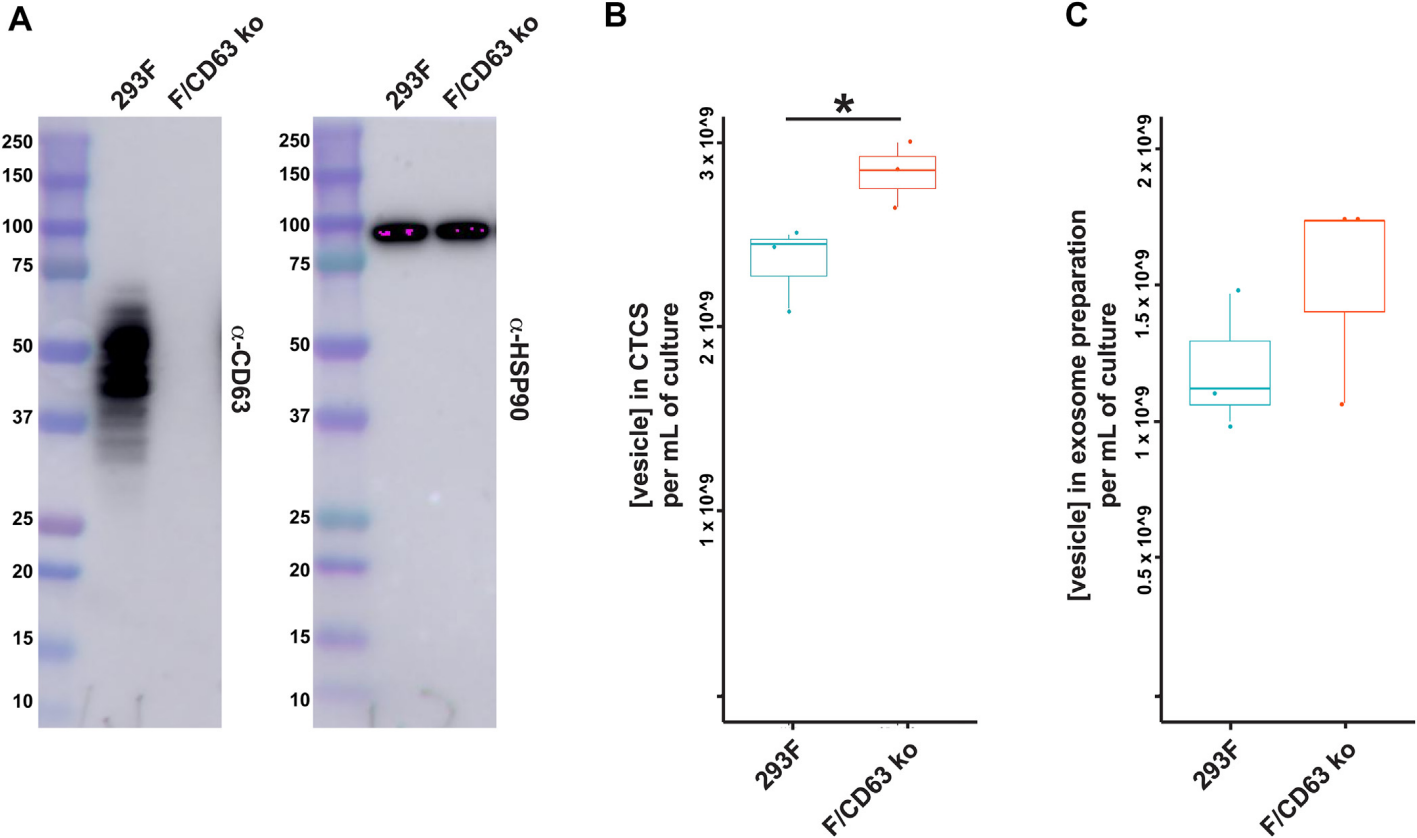
**No decrease in exosome yield by CD63**^**−/−**^**293F cells.***A*, immunoblot analysis of whole cell lysates of WT 293F cells and the F/CD63^−/−^ cell line described previously (137), interrogated with antibodies specific for (*left panel*) CD63 and (*right panel*) HSP90. Molecular weight markers are shown in kDa. *B*, exosome concentration in CTCS samples collected from cultures of WT 293F cells (*left* data, in *blue*) and F/CD63^−/−^ cells *(right data*, in *coral*), as determined by NTA and presented as *box plots*. ∗ denotes *p* <0.05. *C*, exosome concentration in exosome fractions collected from cultures of WT 293F cells (*left data*, in *blue*) and F/CD63^−/−−/−^ cells (*right data*, in *coral*), as determined by NTA, and presented as a box plots. Dots represent individual data points. These experiments were performed three times. CTCS, clarified tissue culture supernatant; NTA, nanoparticle tracking analysis.

Mathieu *et al*. (6) have proposed that the presence of CD63 is not merely a component of endosome-derived exosomes but is in fact their defining component. Others have gone even further and claimed that CD63 is actually required for exosome biogenesis (86). To explore the role of CD63 in the release of exosome-sized vesicles, we took advantage of the F/CD63^−/−^ mutant we recently generated (137) in 293F cells. 293F cells are a derivative of HEK293 cells that can be grown in serum-free, protein-free, chemically defined media, thereby eliminating any potential confounding effects of serum components or exosome-depleted serum on either exosome biogenesis or cell growth (138, 139, 140). This F/CD63^−/−^ cell line carries null mutations on both CD63 alleles (deletion of the intron 4 splice donor site on both of its CD63 alleles (137)) and thus does not express any CD63 protein (Fig. 10*A*).

To determine whether loss of CD63 causes any defect in exosome production, triplicate cultures of WT 293F cells and F/CD63^−/−^ cells were grown in parallel for 3 days, followed by removal of cells by centrifugation (5000*g* for 15 min) and passage of the resulting supernatant through a 200 nm diameter pore-size filter to generate clarified tissue culture supernatants (CTCSs). NTA analysis of these CTCS samples revealed that cells lacking CD63 produced at least as many exosome-sized vesicles as their WT control (Fig. 10*B*) and may actually release slightly more (∼120%; n = 3, *p* = 0.04). It should be noted that measurement of exosome-sized vesicles in these CTCS samples was characterized by low experimental error (4% and 5%, respectively), consistent with the fact that exosome counts in CTCS samples involved few experimental manipulations.

We next purified exosomes from these CTCS samples and again measured exosome yield. Specifically, each CTCS sample was spun at 10,000*g* for 30 min, twice, followed by centrifugation of the resulting supernatant for 2 h at 100,000*g*. Each exosome pellet was resuspended in PBS and exosome yield was quantified by NTA (Fig. 10*C*). These results confirmed that CD63-deficient cells have no defect in the production of exosome-sized vesicles. However, the greater number of experimental manipulations involved in exosome collection resulted in higher percentage errors in these measurements (11% and 28%, respectively) that pushed the higher exosome yield of CD63^−/−^ cells below the range of statistical significance. In addition to these results, the CD63^−/−^ HEK293 cell line (Fig. S2) was assayed for the relative budding of CD9 and CD81 by IB of cell and exosome fractions, which revealed that loss of CD63 has no effect on the budding of these exosome cargo proteins (Fig. S6).

## Discussion

Our current understanding of organelle biogenesis owes much to cargo-based studies that focused on the biogenesis of individual organellar proteins, which provided key insights into how cells make and main the endoplasmic reticulum (141), mitochondria (91, 92, 142), nucleus (98), and peroxisome (94). Here, we applied a similar cargo-based approach to the analysis of exosome biogenesis. Specifically, we identified CD9, CD63, and CD81 as highly enriched exosome cargo proteins and then followed their intracellular sorting and vesicular secretion as a way to explore different hypotheses of exosome cargo protein budding and the biogenesis of exosome-sized vesicles.

### The endosome-dependent hypothesis of exosome biogenesis fails all tests

Using these three highly enriched exosome cargo proteins as test proteins, we explored the relevance of the endosome-dependent hypothesis of exosome biogenesis (3, 7, 47, 110, 143, 144). As summarized in tabular form, our results show that endosome-dependent hypothesis failed to predict or explain the results of every experimental test to which it was subjected (Table 1). In particular, we observed the following:

(i) targeting proteins to endosomes decreases their budding;
(ii) targeting proteins to the plasma membrane increases their budding; and
(iii) KO of purported ‘endosomal regulators’ of exosome biogenesis—Rab27a, Alix, and CD63—had no effect on exosome cargo protein budding or exosome yield.

**Table 1:** The data supports the shared, stochastic model of exosome biogenesis

Experimental result	Endosome-dependent model	Shared, stochastic model	Empirical observations
Endosome-localized cargoes had the highest relative budding	YES	NO	NO
PM-localized cargoes had the highest budding	NO	YES	YES
Redistributing CD63 to the PM decreased its budding	YES	NO	NO
Redistributing CD63 to the PM increased its budding	NO	YES	YES
Redistributing CD9 to the endosome increased its budding	YES	NO	NO
Redistributing CD9 to the endosome decreased its budding	NO	YES	YES
PM-localized and endosome-localized cargoes bud in exosomes of the same size	NO	YES	YES
Redistributing CD63 to the PM increased its cobudding with CD9	NO	YES	YES
Rab27a^−/−^ cells have normal exosome yield & cargo budding	NO	YES	YES
Alix^−/−^ cells have normal exosome yield & cargo budding	NO	YES	YES
CD63^−/−^ cells have normal exosome yield & cargo budding	NO	YES	YES
Levels of CD63, CD9, and CD81 vary by ≥100-fold from one exosome to the next	NO	YES	YES

Taken together, these and other observations raise serious questions about whether the endosome-dependent pathway of exosome biogenesis makes a significant contribution to the production of exosome-sized vesicles by HEK293 cells. Furthermore, the fact that several of these results were also observed in mouse NIH3T3 fibroblasts indicates that these conclusions may apply to mammalian cells generally. If true, it would mean that a great majority of what we know about exosomes has come from the analysis of PM-derived exosomes and not from vesicles that arose from budding at endosome membranes and endolysosomal exocytosis.

### Cells bud exosome cargoes by shared, stochastic mechanism along plasma and endosome membranes

The simplest explanation for the available data is that cells bud exosome cargo proteins by a shared, stochastic mechanism along a spectrum of plasma and endosome membranes, and that most exosomes bud directly from the PM (1). This model is supported by numerous observations (Table 1) but especially by our single-exosome immunophenotyping data. These data, acquired only due to the development of SPIR imaging technology (134), revealed for the first time that individual exosome cargoes vary 100-fold in abundance from one exosome to another, even in antigenically-defined exosome subpopulations. These data also reveal a potential for observational bias in traditional approaches to exosome analysis, which infer the properties of individual exosomes from the averaged properties of billions of exosomes. Specifically, the single-exosome analysis presented here is simply inconsistent with the notion that there are clear and distinct subtypes of exosome-sized vesicles or that one could differentiate them based on the presence of CD9 or CD63. In fact, the only reasonable conclusion that can be drawn from our single exosome immunophenotyping data is that HEK293 cells release a cloud of exosomes, with the composition of each being nearly unique.

### Determinants of exosome heterogeneity

The pronounced degree exosome to exosome compositional heterogeneity established in this study begs the question of how it is generated. The most obvious source of vesicle-to-vesicle heterogeneity is the small size of each exosome, which places a finite limit to the number of molecules that can be packaged in individual vesicles. For example, a vesicle of 50 nm diameter can only accommodate ∼600 surface proteins of ∼4 nm diameter. As a result, each vesicle biogenesis event cannot incorporate anything more than a small subset of the >3000 different exosomal proteins present in just a single cell type (145). Moreover, exosome heterogeneity will be further amplified by (i) nanometer scale heterogeneities that are driven by stochastic (*e.g*., diffusion) and determinative (*e.g*., protein–protein interactions) processes, (ii) micrometer-scale heterogeneities that are driven by differences in protein sorting along plasma and endosome membranes, and (iii) the ∼5-fold variation in exosome radius, which translates to an ∼25-fold variation in exosome surface area and ∼125-fold variation in exosome volume. In addition to these intrinsic drivers of exosome heterogeneity, differences in composition of individual exosomes will be further amplified by temporal fluctuations in RNA and protein expression, cell type–specific difference in RNA and protein expression, and physiologically-triggered changes in exosome cargo protein expression and loading. In short, a single, stochastic mechanism is more than sufficient to generate the pronounced compositional heterogeneity of exosomes observed in this and other reports.

### Relevance to exosome engineering

Exosome engineering is an emerging field, critical to the design, testing, and manufacture of exosomes as vesicle standards, drug delivery vehicles, vaccines, and therapeutics (64, 114, 121, 122, 123, 146, 147, 148, 149). Like all disciplines of engineering, success in exosome engineering requires a solid mechanistic understanding of the factors that determine exosome yield, content, and function. The data presented in this report provide strong evidence that the shared, stochastic hypothesis of exosome biogenesis provides exosome engineers with a much better foundation for the development of exosome-based products, as it is the only hypothesis that explains why targeting proteins to the endosome reduces their budding, why targeting them to the plasma membrane increases their budding, and why KO of Rab27a, Alix, and CD63 have no substantive effect on exosome yield (Table 1). Furthermore, the functional utility of this shared, stochastic hypothesis of exosome biogenesis stands in stark contrast to the endosome-dependent hypothesis of exosome biogenesis, which cannot be used as roadmap for engineering exosome content.

### Lack of requirement for Rab27a, Alix, and CD63

One of the more significant findings of our report was that ablation of the Rab27a, Alix, or CD63 genes had no discernable effect on the budding of exosome cargo proteins or the release of exosome-sized vesicles. These results are at odds with prior claims that these proteins play important roles in exosome biogenesis (77, 82, 85, 86). It is formally possible that the discrepancy between our results and the models claiming important roles for these genes/proteins might reflect cell type–specific differences or compensatory mechanisms, but it is also possible that these genes and proteins are simply not that important to the mechanisms of exosome cargo protein recognition, exosome vesicle budding, or exosome release from cells.

## Experimental procedures

### Plasmids

Plasmids were maintained and amplified in DH10B *Escherichia coli* cells grown in ampicillin-containing LB media. Plasmids were released from cells by base lysis and purified by ion-exchange chromatography. Vectors for expressing WT and mutant forms of CD9 and CD63 were created by PCR amplification of the ORFs, followed by their insertion downstream of the CMV promoter in pcDNA3, with the structure of all amplified segments confirmed by DNA sequencing. For Cas9-mediated gene editing, we created the plasmid pFF, which contains the CMV promoter driving expression of a single long ORF that encodes (i) Cas93xNLS, (ii) a viral 2a peptide, (iii) EGFP, (iv) another viral 2a peptide, (v) the thymidine kinase (tk) from herpes simplex virus (HSV), (vi) another viral 2a peptide, and (vii) the puromycin resistance protein PuroR(123), all flanked by a pair of loxP sites. Into this plasmid, we inserted cassettes that carry two gRNA-expressing genes, with each cassette designed to express gRNAs from the 7sk and H1 promoters that are specific for CD63 (pFF4, targeting 5′-GAGAGCCAGGGGTAGCCCCC-3′ in exon 2), CD9 (pJM1084, targeting GCCCTCACCATGCCGGTCAA in coding exon 1 and 5′-GTCTATATTCTGATCGGAGC-3′ in coding exon 3), Rab27a (pJM1085; targeting 5′-GCCCACTGTTGTGATAAATT-3′ in exon 2 and 5′-ATATTTCTCTGCGAGTGCTA-3′ in exon 4), and Alix (pFF3, 5′-GCGCGCTCGAGACGCTCCTG-3′ and 5’ -GACTGATGGGTACATTGACC-3′) genes, respectively. The primary structures of all PCR-generated segments of all plasmids were confirmed by DNA sequence analysis.

### Cells, culture conditions, and mutation identification

Primary human mammary fibroblasts (ScienCell 7630), primary human dermal fibroblasts (ScienCell 2320), NIH3T3 cells (ATCC CRL-1658), HEK293 cells (ATCC CRL1573), HEK293 gene KO derivatives, 293F cells (Thermo) and the CD63^−/−^ 293F cell line were grown in Dulbecco's modified Eagle's medium (DMEM) supplemented with either 10% fetal bovine serum or 10% exosome-free fetal bovine serum (Gibco A2720801). For measurement of exosome biogenesis by 293F and CD63^−/−^ 293F cells, cells were seeded into Freestyle media (Thermo) at an initial concentration of 1 × 10^6^ cells/ml into shaker flasks and grown for 3 days at 125 rpm in a humidified incubator with 80% CO_2_.

The CD63^−/−^, CD9^−/−/−^, Rab27a^−/−^, and Alix^−/−^ derivatives of HEK293 cells were generated by transfecting HEK293 cells with pFF4, pJM1084, pJM1085, or pFF3 plasmids, followed by selection of transfected cells in media containing puromycin for a period of 7 days. Cells were then seeded individually into 96-well plates, and the resulting SCCs were screened for expression of the targeted protein by either IFM or IB. The CD63^−/−^ 293F cell line was described previously (137).

SCCs that lacked detectable levels of the targeted protein were expanded further, followed by extraction of gDNA from each SCC (Qiagen). Primers flanking each target site (∼150 bp on either side of the target site) were used to amplify ∼300 bp long fragments containing each mutation. These PCR products were separated by gel electrophoresis followed by excision from the gel and insertion into a standard cloning vector. Clones were screened for the presence of gDNA inserts of the correct size, and multiple clones were selected for sequencing. The resulting analysis led to the identification of at least one SCC from each transfection that contained inactivating mutations on all alleles of the targeted gene, with inactivating mutations defined as nonsense mutations, frameshift mutations, or insertions/deletions that precluded the synthesis of more than a small fraction of the ORF and/or splice donor mutations that caused exon skipping and similar consequences. This analysis indicated that HEK293 cells contained only two alleles of the CD63 and Rab27a genes but three alleles of the CD9 gene. Due to the complexity of mutations in the Rab27a^−/−^ cell line, we also extracted RNA from parental HEK293 cells and Rab27a^−/−^ cells (Qiagen), converted this to cDNA (Thermo), and used it as template to amplify, size, and sequence Rab27a cDNA ORFs from both cell lines.

### Mammalian cell transfection

Transfections of HEK293 cells were performed using Lipofectamine 2000 (ThermoFisher) according to the manufacturer’s instructions. NIH3T3-derived cell lines constitutively expressing WT CD63, CD63/Y235A, WT CD9, or CD9/YEVM were generated by transfecting NIH3T3 cells with cognate plasmids, also using Lipofectamine, followed by selection of transgenic cell lines by selection in 400 μg/ml geneticin. SCCs arising from each transfection were then screened to identify cell lines that expressed levels of each protein that were similar to one another and to the levels observed in HEK293 cells.

### Exosome purification

For each biological replicate, 6 × 10^6^ cells were seeded onto 2 × 150 mm dishes in a total volume of 60 ml of DMEM supplemented with 10% exosome-free fetal bovine serum and grown for 72 h. The tissue culture media was collected, spun at 300*g* to remove floating cells or large cell debris, and the supernatant was then spun at 5000*g* for 15 min. The resulting supernatants were then passed through a 200 nm pore size diameter sterile filtration unit to remove microvesicles. For the experiments in Figure 1, Figure 2, Figure 3, Figure 4, the filtrates were then subjected to differential centrifugation involving two 30 min long, 10,000*g* spins, followed by collection of exosomes by centrifugation at 100,000*g* for 2 h. It should be noted that listed g force for these spins represent the midpoint equivalent and that the samples actually experience a spectrum of force that varies in accordance with position in the tube.

For the experiments involving SPIR imaging, the 200 nm pore filtrate was subjected to concentrating filtration across an ∼100 kDa pore size membrane, reducing volume by ∼100-fold and eliminating ∼99% of contaminating proteins. These concentrated vesicle suspensions were then subjected to size-exclusion chromatography using a 10 ml qEVcolumn (Izon).

### IBs

IB analysis was performed by separating cell and exosome lysates by SDS-PAGE, with cell and exosome lysates loaded at a constant ratio, by proportion, of 1:6 cell lysate:exosome lysate. For example, in the experiments presented in Figure 1 we seeded 3 × 10^6^ HEK293 cells onto a 150 mm plate in a volume of 30 ml media. After growing the cells for 3 days, all cells were lysed in 2 ml of SDS-PAGE sample buffer, while all exosomes were purified from the conditioned media, and then lysed in 333 μL of SDS-PAGE sample buffer. Equal volumes of cell and exosome lysate were loaded in adjacent lanes, resulting in 1:6 ratio of cell and exosome fractions by proportion.

Following separation by SDS-PAGE, proteins were transferred to Immobilon membranes (EMDMillipore), which were then incubation with block solution (0.2% nonfat dry milk in Tris-buffered saline with Tween-20 [TBST]) for 2 h, primary antibody solution in block solution overnight, five washes with TBST, secondary antibody solution for 1 h at room temperature (RT), and five washes with TBST. Antigens were then visualized by immersing the membrane in chemiluminescence detection reagents (ECL, MilliporeSigma GERPN2232) and collecting images using an Amersham Imager 600 gel imaging system (GE Healthcare Life Sciences). The resulting digitized IB images were processed in ImageJ (https://imagej.nih.gov/) by converting them to 8 bit grayscale files followed by background subtraction. Measurement parameter and scale were set to integrated density and pixel, respectively. Images were then inverted, bands were delineated using the freehand selection tool, and signal densities were converted to relative protein abundance by multiplying by the dilution factor for each sample. Relative budding was calculated by dividing the relative amount of the protein present in the exosome lysate by the sum of the protein abundance in the cell lysate and the protein abundance in exosome lysate ([amt in exos]/[amt in cells + amt in exos]). Secondary antibodies were obtained from Jackson ImmunoResearch, and primary antibodies were obtained from various sources.

### IFM

IFM was performed on cells grown on cover glasses. Cells were fixed (3.7% formaldehyde in PBS for 15 min), permeabilized (1% Triton X-100 in PBS for 5 min), incubated with primary antibodies in PBS (15 min), washed three times with PBS, incubated with fluorescently labeled secondary antibody and 4′,6-diamidino-2-phenylindole, washed three times with PBS, mounted on glass slides, and visualized by confocal microscopy. Antibodies were diluted in PBS (1:200 dilution for CD63 [clone E-12, #sc-365604, Santa Cruz Biotechnology], 1:200 dilution for CD9 [clone H19a, #312102, Biolegend], 1:1000 dilution of fluorescein (FITC) AffiniPure goat antimouse IgG (H + L) [#115–095–003 Jackson Laboratory]). Confocal images were acquired using a Zeiss AxioObserver inverted microscope with LSM700 confocal module and 63×, 1.4 aperture AxioPlan objective. Images were acquired using Zen software (Zeiss), converted to tiff files, and imported into Adobe Photoshop and Illustrator to create final images. Standard immunofluorescence imaging of NIH3T3 cells was performed at RT on a BH2-RFCA microscope (Olympus) equipped with an Olympus S-plan Apo 63 × 0.40 oil objective and a Sensicam QE (Cooke) digital camera using IPLab 3.6.3 software (Scanalytics, Inc). Tiff images were imported into Adobe Photoshop and Illustrator to create final images.

### SPIR imaging and coupled SPIR imaging & immunofluorescence

Each exosome sample was diluted 10-fold in SPIR incubation buffer (50 mM Hepes, 150 mM NaCl, and 0.05% Tween-20, pH 7.3). Thirty-five microliters of each sample were then incubated on the ExoView Tetraspanin Chip (EV-TC-TTS-01) placed in a sealed 24-well plate for 16 h at RT. Each chip was then washed on an orbital shaker once with PBST (PBS supplemented with 0.05% Tween-20) for 3 min, then washed three additional times with PBS for 3 min each. Chips were then incubated with one or more of Alexa-55-labeled anti-CD81, Alexa-488-labeled anti-CD63, and Alexa-647anti-CD9 antibodies in PBST supplemented with 2% bovine serum albumin in a volume of 250 μl for 2 h at RT without shaking. Each chip was then washed once with PBST, three times with PBS, once in filtered deionized water, and then dried at RT for 1 h. The chips were then imaged with the ExoView R100 reader using the ExoScan 2.5.5 acquisition software (Nanoview Biosciences). The resulting size and fluorescence intensity information for each individual exosome was exported to Excel for statistical analyses. Fluorescence values are reported in arbitrary units.

### Exosome analysis by NTA and RPS

Exosomes were interrogated for size and concentration by dilution into filtered PBS (100 nm pore size), followed by (i) NTA analysis on a Particle Metrix Zetaview Twin PMX-220 according to the manufacturer’s instructions, and (ii) RPS analysis on a Spectradyne nCS-1 according to the manufacturer’s instructions.

### Data analysis and presentation

Statistical analysis involved calculation of averages and standard error of the mean, with pairwise differences evaluated for likelihood of null hypothesis using Student’s *t* test, or ANOVA for experiments evaluating more than two sample sets. Histograms and scatter plots were generated using Excel. Images were imported into Adobe Photoshop and figures were assembled in Adobe Illustrator. Image data were adjusted for brightness only.

## Data availability

All data is contained within this article.

## Supporting information

This article contains supporting information (150).

## Conflict of interest

The authors declare that they have no conflicts of interest with the contents of this article.
