# Supplementary material for: A shared, stochastic pathway mediates exosome protein budding along plasma and endosome membranes
Source: J Biol Chem. 2022 Aug 18;298(10):102394. doi: 10.1016/j.jbc.2022.102394 (PMC9512851; doi:10.1016/j.jbc.2022.102394)
Supplement: Supporting Information [file mmc1.pdf]

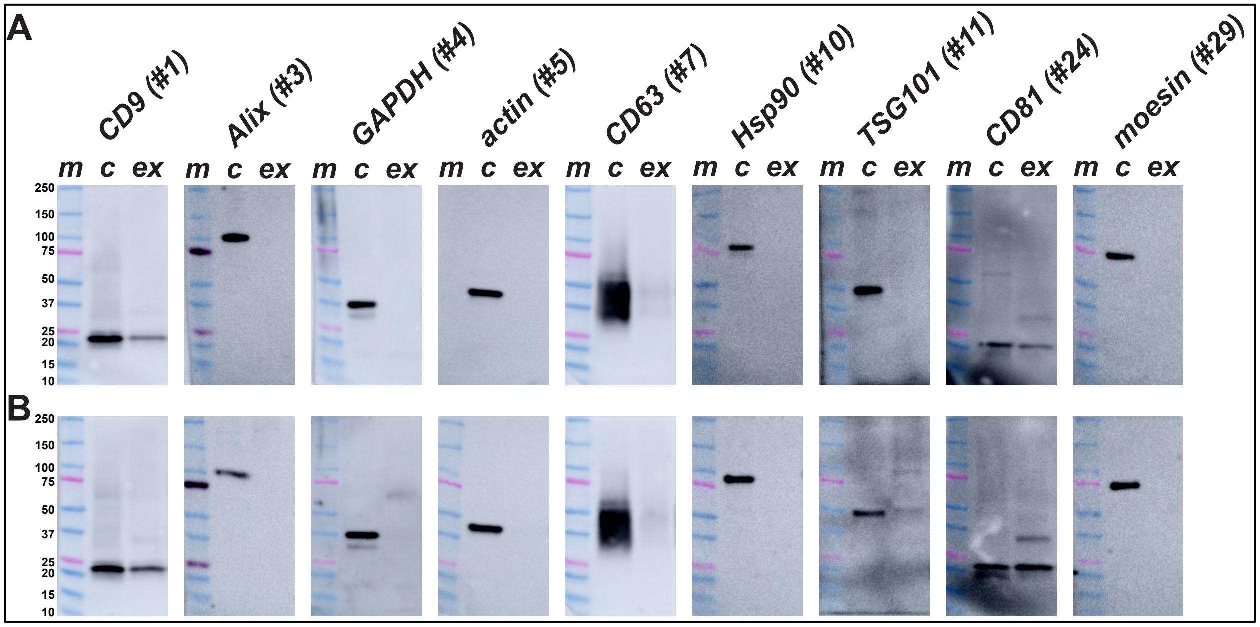

Figure S1. CD9, CD63, and CD81 are highly-enriched proteins of human primary fibroblast exosomes. Cell and exosome fractions were collected from cultures of (**A**) primary human mammary fibroblasts and (**B**) primary human dermal fibroblasts, then lysed and processed for immunoblot using antibodies specific for eight reported exosomal proteins. m, marker; c, cell lysate; ex, exosome lysate. Molecular weights of size markers are listed to the left. Numbers in parentheses refer to the rank on the exocarta.org(Keerthikumar *et al.*, 2017) list of 'exosome marker proteins' as of Jan 1 2022. These experiments were performed twice.

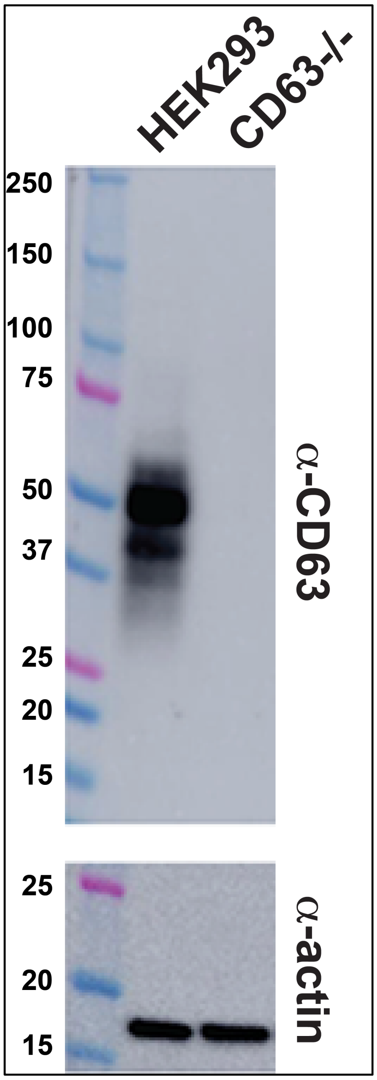

Figure S2. Immunoblot analysis of a CD63$^{-/-}$ derivative of HEK293 cells. Equal numbers of WT HEK293 cells and CD63_ko_SCC4 (HEK29) cells were lysed in SDS-PAGE sample buffer, separated by SDS-PAGE, and processed for immunoblot using antibodies specific for (upper panel) CD63 and (lower panel) actin. m, marker; c, cell lysate; ex, exosome lysate. Molecular weight markers are, from top, in kDa, 250, 150, 100, (pink) 75, 50, 38, (pink) 25, and 20 for the anti-CD63 blot, and, from top, 75, 50, and 38 for the anti-actin blot. Sequence analysis of genomic DNA surrounding the Cas9-targeted site in exon 2 of the CD63 gene revealed that allele #1 contained a 113 bp insertion in exon 2 that shifts the reading frame after Gly46 and results in premature termination, while allele #2 contained a 1 bp deletion in exon 2 that also shifts the reading frame after Gly46 and results in premature termination. These experiments were performed three times.

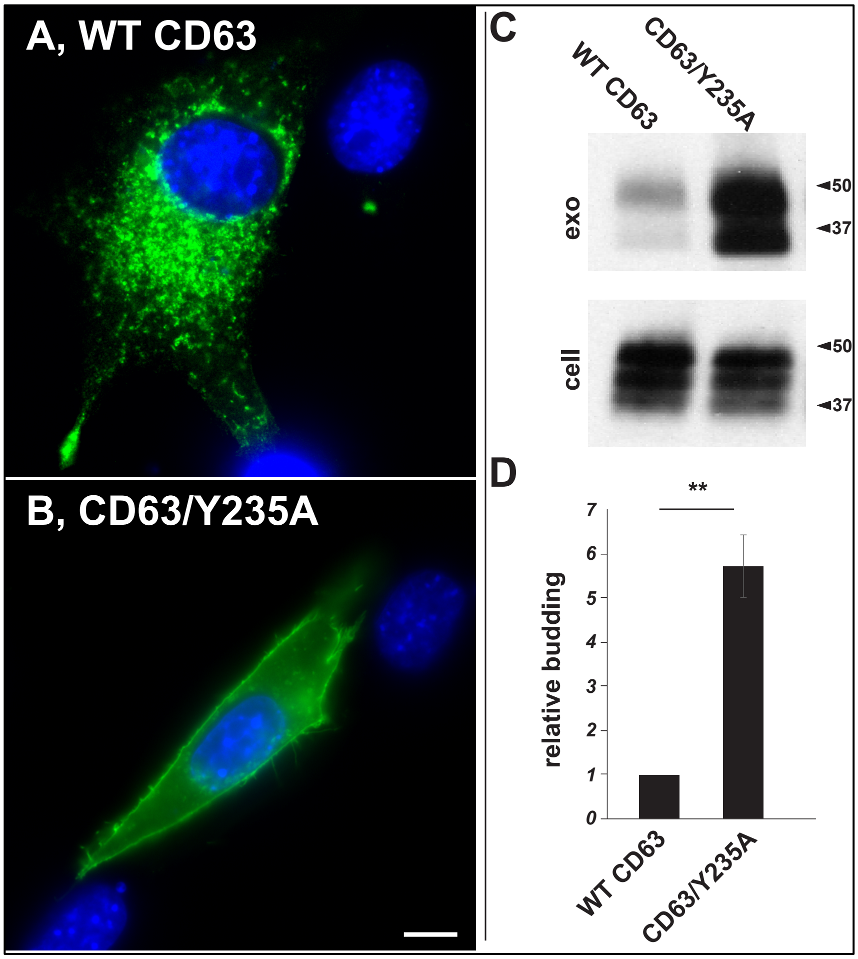

Figure S3. Redirecting CD63 to the plasma membrane of NIH3T3 cells increases rather than decreases its vesicular secretion. (**A**, **B**) Confocal fluorescence micrographs of NIH3T3 cells that had been transfected with plasmids designed to express either (A) WT human CD63 or (B) human CD63/Y235A, then fixed, permeabilized, stained with DAPI, and then processed for immunofluorescence microscopy using a monoclonal antibody specific for human CD63. Bar, 10 µm. (**C**) Immunoblot of cell and exosome fractions collected from NIH3T3 cells expressing either WT human CD63 or human CD63/Y235A, probed with a monoclonal antibody specific for human CD63. (**D**) Bar graph of the relative budding of CD63 and CD63/Y235A, with bar height representing the average and error lines denoting the standard error of the mean (s.e.m.), normalized to that of CD63. n = 6; ** denotes a p value <0.005. These experiments were performed six times.

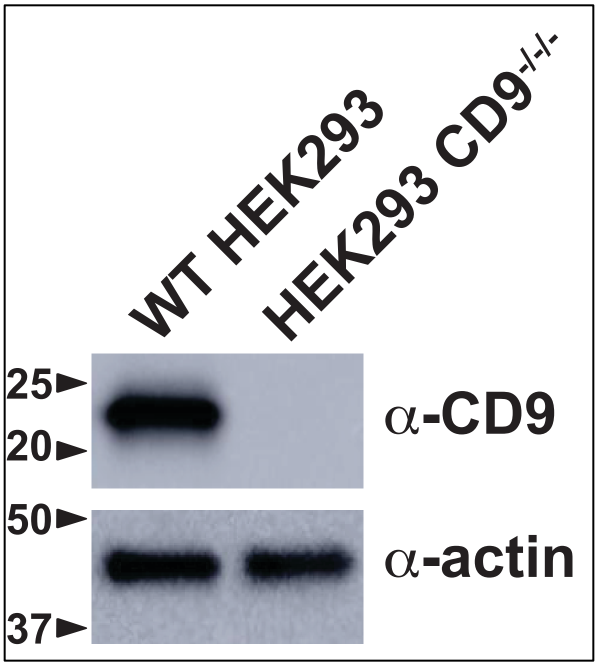

Figure S4. Immunoblot analysis of a CD9-/-/- derivative of HEK293 cells. Equal numbers of WT HEK293 cells and CD9_ko_2 (HEK29) cells were lysed in SDS-PAGE sample buffer, separated by SDS-PAGE, and processed for immunoblot using antibodies specific for (upper panel) CD63 and (lower panel) actin. Sequence analysis of genomic DNA surrounding the Cas9-targeted sites in exon 1 and exon 3 of the CD9 gene revealed that allele #1 contained a 2 bp insertion in exon 1 that shifted the reading frame after Val3 (of the 228 amino acid-long protein), allele #2 carried a deletion of all sequences between the exon 1 and exon 3 target sites, also shifting the reading frame after Val3, and allele #3 contained a 4 bp insertion in exon 3 that shifted the reading frame after Gly65. These experiments were performed three times.

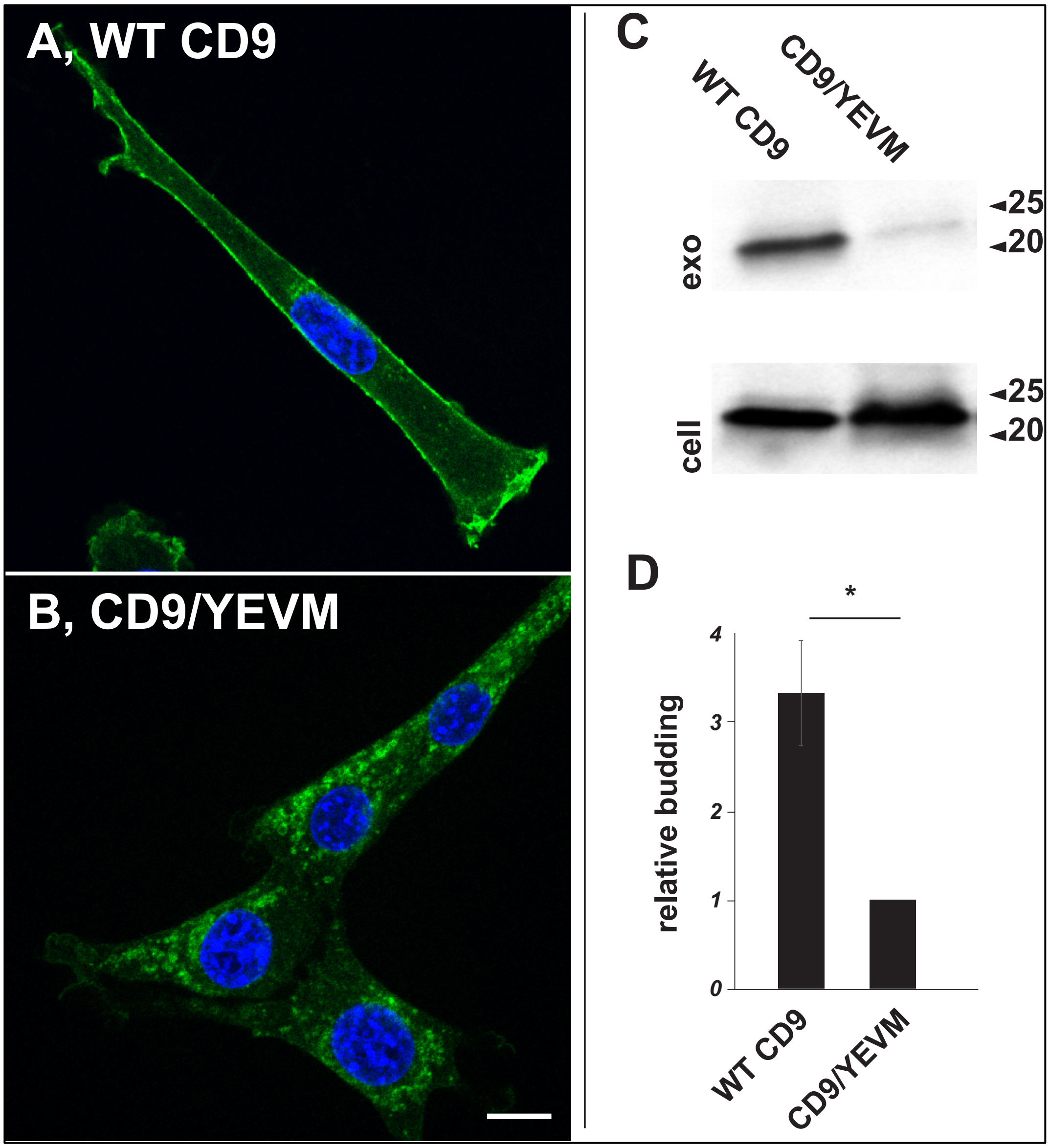

Figure S5. Redirecting CD9 to the endosome of NIH3T3 cells decreases rather than increases its vesicular secretion. (**A**, **B**) Confocal fluorescence micrographs of NIH3T3 cells that had been transfected with plasmids designed to express either (A) WT human CD9 or (B) human CD9/YEVM, then fixed, permeabilized, stained with DAPI, and then processed for immunofluorescence microscopy using a monoclonal antibody specific for human CD9. Bar, 10 µm. (**C**) Immunoblot of cell and exosome fractions collected from NIH3T3 cells expressing either WT human CD9 or human CD9/YEVM, probed with a monoclonal antibody specific for human CD9. (**D**) Bar graph of the relative budding of CD9 and CD63/YEVM, with bar height representing the average and error lines denoting the standard error of the mean (s.e.m.), normalized to that of CD9/YEVM. n = 6; * denotes a p value <0.05. These experiments were performed six times.

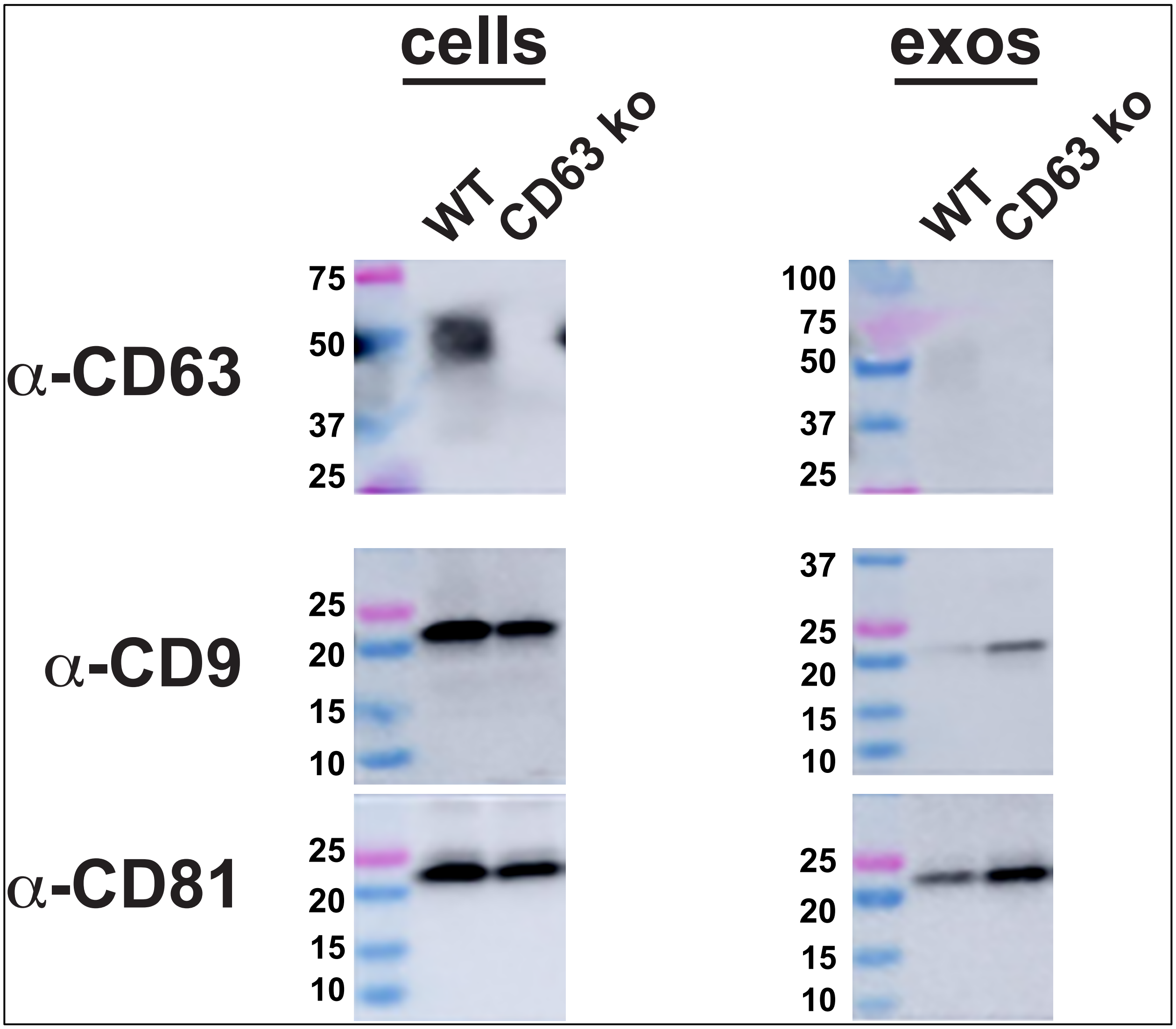

Figure S6. Disruption of CD63 has no effect on exosome biogenesis. HEK293 cells and the CD63_ko_SCC4 (HEK29) cell line were grown in DMEM containing 10% exosome-depleted FBS for 3 days, followed by collection of cell and exosome fractions. Cells and exosomes were interrogated by immunoblot (loaded at a 1:6 ratio by proportion of the total samples) using antibodies specific for CD63, CD9, and CD81. Sizes of molecular weight markers are noted to the left of each panel. These experiments were performed three times.
